# Peculiar Porous Aluminum Oxide Films Produced via Electrochemical Anodizing in Malonic Acid Solution with Arsenazo-I Additive

**DOI:** 10.3390/ma14175118

**Published:** 2021-09-06

**Authors:** Alexander Poznyak, Gerhard Knörnschild, Anatoly Karoza, Małgorzata Norek, Andrei Pligovka

**Affiliations:** 1 Department of Electronic Technology and Engineering, Belarusian State University of Informatics and Radioelectronics, 6 Brovki Str., 220013 Minsk, Belarus; poznyak@bsuir.by; 2 Research and Development Laboratory 4.10 “Nanotechnologies”, Belarusian State University of Informatics and Radioelectronics, 6 Brovki Str., 220013 Minsk, Belarus; 3 Department of Metallurgy, Federal University of Rio Grande do Sul, Porto Alegre 90040-060, Brazil; Gerhard.Hans@ufrgs.br; 4 Centre for Analytical and Spectral Measurements, B.I. Stepanov Institute of Physics of the National Academy of Sciences of Belarus, 68-2, Nezavisimosti Ave., 220072 Minsk, Belarus; a.karoza@ifanbel.bas-net.by; 5 Institute of Materials Science and Engineering, Faculty of Advanced Technologies and Chemistry, Military University of Technology, 2 Kaliskiego Str., 00-908 Warsaw, Poland; malgorzata.norek@wat.edu.pl; 6 Department of Micro- and Nanoelectronics, Belarusian State University of Informatics and Radioelectronics, 6 Brovki Str., 220013 Minsk, Belarus

**Keywords:** alumina, valve metal, 3-(2-arsonophenyl)azo-4,5-dihydroxy-2,7-naphthalenedisulfonic acid disodium salt, complex compound, chelate complex, volume growth factor, anodizing efficiency

## Abstract

The influence of arsenazo-I additive on electrochemical anodizing of pure aluminum foil in malonic acid was studied. Aluminum dissolution increased with increasing arsenazo-I concentration. The addition of arsenazo-I also led to an increase in the volume expansion factor up to 2.3 due to the incorporation of organic compounds and an increased number of hydroxyl groups in the porous aluminum oxide film. At a current density of 15 mA·cm^−2^ and an arsenazo-I concentration 3.5 g·L^−1^, the carbon content in the anodic alumina of 49 at. % was achieved. An increase in the current density and concentration of arsenazo-I caused the formation of an arsenic-containing compound with the formula Na_1,5_Al_2_(OH)_4,5_(AsO_4_)_3_·7H_2_O in the porous aluminum oxide film phase. These film modifications cause a higher number of defects and, thus, increase the ionic conductivity, leading to a reduced electric field in galvanostatic anodizing tests. A self-adjusting growth mechanism, which leads to a higher degree of self-ordering in the arsenazo-free electrolyte, is not operative under the same conditions when arsenazo-I is added. Instead, a dielectric breakdown mechanism was observed, which caused the disordered porous aluminum oxide film structure.

## 1. Introduction

The current area of nanotechnology, which is rapidly developing at present, is the creation of functional materials using porous and tubular anodic oxides of aluminum and other valve metals [1,2,3,4,5,6,7,8,9,10,11]. The ratio of simultaneously occurring self-organized processes of metal oxidation with the formation of oxide and dissolution plays an important role in the formation of porous and tubular anodic oxides of valve metals. Different kinds of metal dissolution during anodic oxidation of aluminum and other valve metals lead to the formation of oxide films with different morphologies: barrier layers, quasi-regular porous layers, high degree self-ordering porous layers as well as tubular and nanocomposite structures [6,12,13,14,15,16,17,18,19,20,21,22,23,24,25,26,27,28,29,30,31,32,33,34]. The anodizing electrolyte is, aside from other process parameters, the main factor that determines this morphology. Barrier layers, which are interesting because of their dielectric properties [35], are formed in baths where oxide dissolution is slow [36]. The dissolving power of the electrolyte can be varied in two approaches: the introduction of ligands forming stable complex compounds with the ions of the anodized metal and the change in the pH value of the electrolyte [33], and the dissolving power of the electrolyte can be easily controlled by introducing small additions of fluoride ions that form stable complex compounds with the ions of the listed metals. Due to the oxide chemical properties of most refractory metals (titanium, hafnium, niobium, tantalum, tungsten, vanadium, and zirconium) [37], the dissolving power of the electrolyte can be easily controlled by introducing small additions of fluoride ions that form stable complex compounds with the ions of the listed metals [38]. Similarly, enhanced dissolution was used as an explanation given for TiO_2_ nano-tube formation in fluoride–glycerol electrolytes, in this case, the dissolution of a fluoride-rich layer, which separates the nano-tubes [39].

Porous aluminum oxide films (PAOFs) are widely used for surface treatments, where the porous structure enables coloring for esthetic purpose or sealing in order to improve corrosion protection. Sulfuric, chromic, oxalic, and phosphoric acids are the most widely used baths for this purpose [40]. Along with these traditional applications, the cellular-porous structure of PAOF provides ample opportunities for controlled variation of its morphological parameters, making it possible to create a wide range of functional materials [7,26,41,42,43,44]. For this, unconventional electrolytes are being actively investigated [31,45,46,47,48,49,50,51,52,53]. The second approach (described in the previous paragraph) is traditionally used to form the PAOF. A sufficiently high degree of acid dissociation (i.e., a low pKa), seems to be necessary in order to obtain a PAOF, since it allows for the production of electrolytes with a low pH value and high dissolving power. As it is known [37], the chemical activity of aluminum oxide increases sharply with a significant deviation of pH from neutral. In [50], the authors showed that a barrier film is formed in glutaric acid with pKa = 4.13, while a PAOF is formed in ketoglutaric acid, where the additional keto group has an electron withdrawing effect that lowers pKa to 1.85. More generally, the electron withdrawing effect of keto and/or hydroxyl groups, which lowers pKa, favors PAOF, and according to [50], at the same time favors complexation of Al^3+^ ions. At the same time, there is an opinion that chemical dissolution, however, is not the mechanism that promotes Al_2_O_3_ dissolution at the pore ground during anodizing, since it is orders of magnitude too slow [54]. Instead, field assisted dissolution and direct ejection of Al^3+^ ions into the electrolyte are thought to be the processes that lead to PAOF formation [55,56]. Mass transport from the pore ground toward the cell walls occurs due to compressive stress in the oxide [57], forming hexagonal cells with a concave metal oxide interface [58]. The porous structure becomes highly self-ordered, when cell formation is guided by a hexagonal pattern formed at the metal surface as the first step of a two-step [59] or three-step [60,61] anodizing process. Nielsch et al. in [62] observed that highly ordered PAOF were obtained in different electrolytes when the porosity was close to 10%. According to the authors, this corresponds to a volume expansion factor of 1.2. Recently, ordered porous structures in malonic acid (MA) were found to grow under the mechanism that holds the volume expansion factor constant [51]. Ono et al. in [63] showed that high currents and high electric fields, but without reaching a critical voltage threshold for breakdown, are favorable conditions for highly ordered PAOF.

Variations of PAOF parameters such as interpore distances, pore diameters, and porosity were obtained by the use of different acids: inorganic such as selenic [52,64,65], nitric [45,46], sulfuric [66,67], and phosphoric [66,68,69], but mostly organic such as oxalic [66,68], tartaric [63,70], malic [71], MA [63,72,73,74] and by organic additives like ethanol [75], ethylene glycol [76], and polyethylene glycol [77]. In some of the mixed anodizing electrolytes such as oxalic acid with ethylene glycol [78], a more radical change in the film morphology from porous to tubular was observed.

The composition of the electrolyte for anodizing aluminum [79,80,81], and the modes of the experiment [23,81,82] significantly affect the properties and composition of the PAOF. So far, there is no clear idea on how these acids and organic additives interact with the anodizing process and how this leads to the observed modifications in PAOF growth. Norek et al. in [78] suggested that the incorporation of soluble C_2_O_4_^2−^ and COO^−^ ions into the oxide framework could lead to enhanced oxide dissolution and preferred cleavages along the cell boundaries [79,80,81]. From the point of view of additional possibilities for controlling the PAOF composition, the processes of obtaining PAOF in electrolytes, which contain complex compounds [83,84,85], are of great interest.

Despite the importance of metal dissolution for all film morphologies, dissolution by chelating or generally by complexing agents during anodizing has, so far, not been explicitly studied. It should be mentioned that inorganic anodic acids such as orthophosphoric [86], and organic [47,87,88,89] such as citric, tartaric, and oxalic acids have a pronounced tendency to form soluble complex compounds with the aluminum ion [38]. Thus, in fact, both approaches are combined: dissolution due to a change in the pH of the electrolyte, combined with the ability of the acid residue ions to act as ligands. As shown earlier [81], it is also possible to specially introduce additives containing ligands that form stable soluble complex compounds with aluminum [38], which leads to a significant change in both the nature of the anodic oxidation process and the morphology of anodic oxide. Similar studies were later carried out using the organic reagent 3-(2-arsonophenyl)azo-4,5-dihydroxy-2,7-naphthalenedisulfonic acid disodium salt (arsenazo-I), which forms extremely stable chelating complexes with the Al^3+^ ion and some others [38]. Arsenazo-I and its derivates are known for their chelating action and have been used for a long time for analytical purposes [38,90,91,92,93,94]. The first scattered brief reports on the results of such studies appeared in the works of [95,96].

In the present work, anodizing of aluminum in MA with the addition of arsenazo-I was studied. As shown above, the introduction of complexing additives into the anodizing electrolyte or a change in the nature of the electrolyte significantly affects the anodizing process nature and the properties of the resulting anodic oxides, which can be used to create new materials and electronic devices. Data were obtained on galvanostatic anodizing of high purity aluminum in 0.6 M MA containing up to 4.0 g·L^−1^ arsenazo-I, and features of the formation process, composition, structure, and morphology of PAOF were studied.

## 2. Materials and Methods

### 2.1. Film Preparation

**Figure 1:**
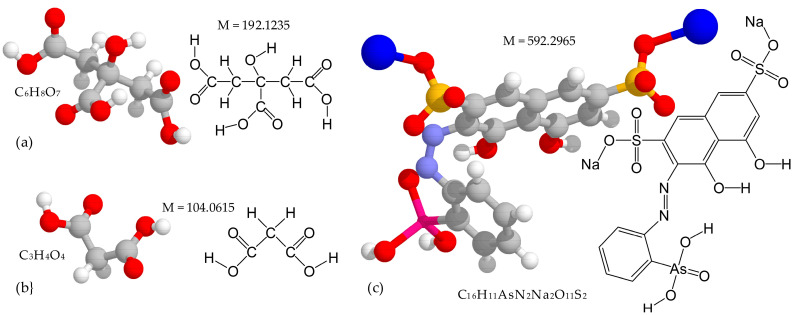
Chemical structure of (**a**) citric and (**b**) malonic acids and (**c**) arsenazo-I.

Aluminum foils used for anodizing had a purity of 99.99% and a thickness of 10.5 μm. The procedure for sample preparation and anodizing tests was basically the same as the one described recently [48,51]. The foils were cleaned in distilled water and dried with air. The foils were applied without further chemical pretreatment. An area of 1–4 cm^2^ was exposed to the solution. The rest of the sample surfaces were electrically isolated by previous anodizing in 1% citric acid (Sigma-Aldrich, Darmstadt, Germany) with a maximum voltage of 290 V, which was held until the current density fell below 5% of its initial value. A part of the sample protected by the barrier oxide served to provide electrical contact with a power source and prevent a meniscus effect. The chemical structure of citric acid is shown in Figure 1a.

The free surface was galvanostatically anodized from both sides, simultaneously, in 0.6 M MA (Sigma-Aldrich, Darmstadt, Germany) with the addition of arsenazo-I (Reachem, Moscow, Russia). The chemical structures of MA and arsenazo-I are shown in Figure 1b,c, respectively. The 3D structures shown of the used chemicals make it possible to estimate the relative sizes of the molecules, clearly demonstrate their spatial structure and explain, in particular, the ability of arsenazo-I to act as a ligand in the composition of very stable complex compounds with many metal cations. The arsenazo-I concentration varied between 0.1 g·L^−1^ and 4.0 g·L^−1^. For the experiments, two solutions were prepared: 0.6 M MA solution and solution containing 4 g·L^−1^ arsenazo-I in 0.6 M MA solution. MA solutions with the addition of arsenazo-I of intermediate concentrations were prepared by mixing the required volumes of the listed solutions. Volume *V_el_* of the anodizing electrolyte was about 40 mL, which was determined by the formula

(1) $$V_{el}=V_{MA}+V_{Ars}$$

where *V_MA_* and *V_Ars_* are the volume, mL, of 0.6 M MA and 0.6 M MA with an arsenazo-I additive 4.0 g·L^−1^, respectively.

Arsenazo-I additive concentration in solution *C_Ars_* was calculated using the formula

(2) $$C_{Ars}=\frac{C_{0,Ars}\cdot V_{Ars}}{V_{el}}$$

where *C*_0,*Ars*_ is the arsenazo-I concentration, equal 4.0 g·L^−1^.

For comparative analysis, anodizing was performed in MA without additives at current densities of 15 and 200 mA·cm^−2^. In addition, when considering and analyzing the work results, the results of an experiment series on anodizing aluminum in 0.6 M MA were partially used without the presence of any additives [48,51]. The samples were positioned in the middle between two parallel counter electrodes, in order to obtain a homogeneous electric field, while they were completely anodized from both sides. During all the tests, the electrolyte was stirred using a magnetic stirrer, and temperature (294 K, with warming less than 2 K during the test) was kept constant.

The major part of the tests was performed at anodizing current densities of 15, 100, and 200 mA·cm^−2^, respectively. Electric anodizing modes were set and controlled using a potentiostat П-5827 M (Measuring Instruments Plant, Gomel, USSR). Programmable digital multimeters 34470 A (Keysight Technologies Inc., Santa Rosa, CA, USA) were used to record the voltage–time responses, controlled by a PC with homemade software written in LabVIEW 2018. The completion of the oxidation of the whole foil was determined from the steep voltage rise that was observed when the metal was almost completely consumed. From the measurement of the initial thickness of the aluminum foil *h_Al_* and the PAOF thickness *h_PAOF_* after complete anodizing of the foil, the volume expansion factor *K_V_* was determined by micrometer measurements and selectively verified by scanning electron microscope (SEM). To determine both the starting aluminum foil and the resulting PAOF thickness, a digital micrometer Micromar 40 EWR (Mahr Inc., Providence, RI, USA) was used.

The volume expansion factor *K_V_* was calculated as

(3) $$K_{V}=\frac{h_{PAOF}}{h_{\mathrm{Al}}}$$

The loss of Al dissolved to the electrolyte during anodizing was analyzed by inductively coupled plasma atomic emission spectroscopy (ICP-AES) using the inductively coupled plasma atomic emission spectrometer IRIS Intrepid II, XDL (Thermo Fisher Scientific Inc., Waltham, MA, USA). The concentration of Al^3+−^*C*_Al_, μg·mL^−1^, was determined from the intensities of emission at 394.4 and 396.1 nm. The mass loss per PAOF surface area, *m_diss_*, μg·cm^−2^, was calculated by:

(4) $$m_{diss}=\frac{C_{\mathrm{Al}}\cdot V_{el}}{S_{ox}}$$

where *S_ox_* is the surface area, cm^2^, of the PAOF.

The average current density of aluminum dissolution *j_diss_*, mA·cm^−2^, is given by:

(5) $$j_{diss}=\frac{3\cdot m{}_{diss}\cdot F}{M_{\mathrm{Al}}\cdot \tau_{anod}}$$

where *F* is the Faraday constant (96,484.56 C·mol^−1^). *M*_Al_ is the aluminum molar mass (26.98154 g·mol^−1^) and τ*_anod_*, s, is the time for complete anodizing.

The current efficiency η*_F_* was determined from the theoretical charge *Q_ox,th_*, C·cm^−2^, needed to oxidize the whole aluminum sheet, according to

(6) $${\mathrm{Al}}^{0}-3e\to {\mathrm{Al}}^{3+}$$

and from the electric charge *Q_ox,real_*, C·cm^−2^, really needed to complete anodizing of the foil

(7) $$Q_{ox,real}=j_{a}\cdot \tau_{anod}$$

where *j_a_* is the anodizing current density, mA·cm^−2^, obtained from the voltage–time responses during the galvanostatic tests:

(8) $$\eta_{F}=\frac{Q_{ox,th}}{Q_{ox,real}}$$

The efficiency of PAOF formation η*_PAOF_* is calculated from dissolved aluminum by:

(9) $$\eta_{PAOF}=1-\frac{j_{diss}}{j_{a}}$$

### 2.2. Characterization and Measurements

Thickness, morphology, structure, and composition of the PAOFs were examined by scanning electron microscopy (SEM), X-ray diffraction (XRD), Fourier transform infrared (FTIR) spectroscopy, and X-ray photoelectron spectroscopy (XPS), respectively.

The surfaces and sections of samples were observed with a Hitachi S-806 field-emission SEM (Hitachi, Ltd., Marunouchi, Chiyoda-ku, Tokyo, Japan) operated at 20 kV. Before SEM observation, some anodized specimens were mechanically broken to obtain cross-sections, and a gold layer, with a thickness of about 3 nm, was evaporated onto the fractures and surfaces of all PAOFs.

Investigations of the PAOF composition were carried out by X-ray powder diffraction performed on a diffractometer DRON-3 (Bourevestnik, JSC, St. Petersburg, Russia) connected to a personal computer, with Cu-Kα-radiation with a graphite filter. For convenient handling, the brittle PAOF samples were glued to a glass substrate with BF-2 glue, and then fixed in a holder.

FTIR spectra of PAOFs were registered with a Thermo Nicolet Nexus IR-spectrometer (Thermo Fisher Scientific Inc., Waltham, MA, USA) with a wave number range of 4000 cm^−1^ to 400 cm^−1^ and a resolution of 2 cm^−1^, after 128 scans using a Deuterated Tri Glycine Sulfate (DTGS) detector.

Photoelectron spectra were obtained on an X-ray photoelectron spectrometer ЭC 2402 (Production of the Experimental Plant of Scientific Instruments of the USSR Academy of Sciences, Chernogolovka; Production Association “Nauchpribor”, Orel, USSR) using X-ray radiation from the Kα-line of Mg (hν = 1253.6 eV). When identifying the photoelectron spectra, the binding energies of the main electrons C1s, O1s, Al2s, Al2p, S2p, and As3d were taken into account. To calibrate the spectra, the C1s line from surface hydrocarbon contaminants was used, for which the binding energy was taken to be 284.8 eV.

### 2.3. Data Operation

Mathematical processing and graphic visualization of experimental data were carried out using programs and software packages:

ChemWindow (Bio-Rad Laboratories Inc., Sadtler Division, Grand Junction, CO, USA);
Stanford Graphics Version 3.0 (Visual Numerics Inc., Houston, TX, USA);
OriginPro 2018 (OriginLab Corporation, Northampton, MA, USA); and
Microsoft Office Excel 2019 (Microsoft Corporation, Redmond, WA, USA).

## 3. Results and Discussion

### 3.1. Anodizing Behavior

**Figure 2:**
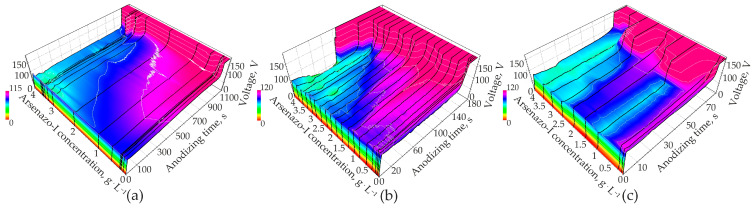
Dependences of anodic voltage on time and concentration of arsenazo-I additive for current densities (**a**) 15, (**b**) 100 and (**c**) 200 mA·cm^−2^.

An overview of the anodic voltage time dependences on the arsenazo-I additive concentration for the three studied values of the anodic current densities are shown in Figure 2 in the form of surfaces modeled on the basis of the corresponding curves.

**Figure 3:**
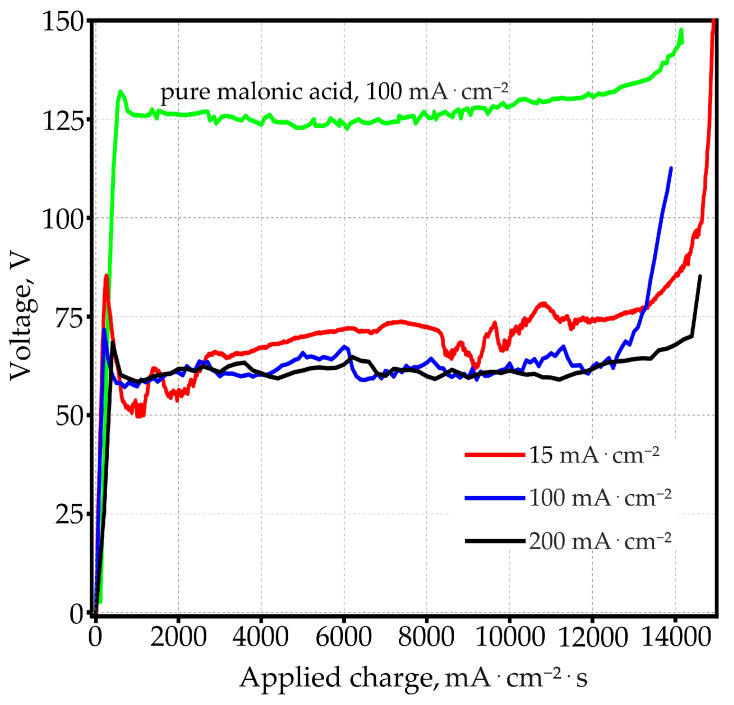
Dependence of anodic voltage on applied charge during galvanostatic anodizing in 0.6 M malonic acid pure and with 3.5 g·L^−1^ arsenazo-I additive at anodic current densities 15, 100, and 200 mA·cm^−2^.

Voltage transients for the three current densities studied in the present work are shown in Figure 3. The curves were measured in 0.6 M MA with the addition of 3.5 g·L^−1^ of arsenazo-I. For a more correct comparison of the anodizing sample behavior formed at different anodic current densities, the time axis was normalized by multiplication with the galvanostatic applied current density:

(10) $$Q_{ox}=j_{a}\cdot \tau_{anod}$$

where *Q_ox_* is the applied charge, mA·cm^−2^·s (mC·cm^−2^).

Thus, the abscissa axis is now actually graduated in units of the charge expended at each moment of time for the anodic oxidation of the aluminum foil. In order to visualize the changes introduced by arsenazo-I a curve in pure 0.6 M MA was added. It can be seen that arsenazo-I considerably lowers the anodizing voltages when compared at the same current density. With arsenazo-I the curves show a pronounced voltage overshot at the end of the linear voltage rise. Another detail that differs in curves with and without arsenazo-I is the unusually strong voltage oscillation in the stationary part of the process. It is also unusual that with higher applied anodizing current, the stationary anodizing voltage tends to become lower, unlike the behavior observed under the same conditions in an electrolyte free of arsenazo-I, described in a recently published work [51]. The steep voltage rise at the end occurs since the aluminum foil is completely anodized from both sides, making the metal disappear at the end.

**Figure 4:**
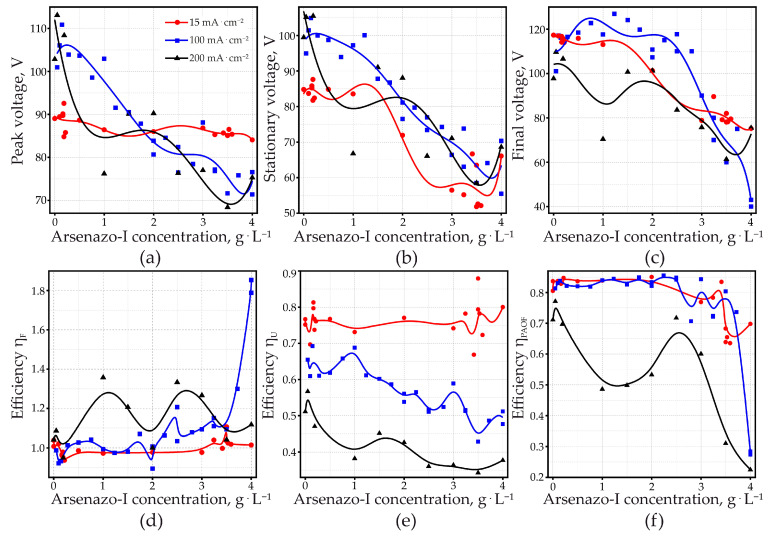
(**a**) Dependence of peak voltage, (**b**) stationary anodic voltage (showing the value measured at 50% of total applied charge), (**c**) and final voltage on arsenazo-I concentration for Al in 0.6 M malonic acid. (**d**) Current efficiency η*_F_*, (**e**) anodizing efficiency, obtained from dU/dτ η*_U_*, and (**f**) anodizing efficiency, obtained from dissolved Al^3+−^, η*_PAOF_*, as a function of arsenazo-I concentration. Galvanostatic anodizing with 15, 100, and 200 mA·cm^−2^, respectively.

Increasing the arsenazo-I concentration generally leads to a decrease in the peak, stationary, and final anodizing voltages (Figure 4a–c). At 15 mA·cm^−2^, the influence of arsenazo-I was approximately linear, while at 100 and 200 mA·cm^−2^, data were more scattered. Since the influence of arsenazo-I on the conductivity of the electrolyte is small, the decreasing anodizing voltage at constant current density indicates an increase in conductivity of the PAOF, most probably due to a higher defect concentration as a consequence of arsenazo-I incorporation into the PAOF. The correlation between anodizing voltage and incorporation of bath components has already been observed with respect to anions from the acid. Particularly in the case of sulfuric acid, anodizing contamination with sulfate is high [97] and at the same time, the anodizing voltage is much lower than for less contaminated films formed in various organic acid electrolytes.

### 3.2. Current Efficiency

The current efficiency η*_F_*, which theoretically should not exceed 1 (100%), characterizes the amount of charge consumed for the aluminum oxidation and the implementation of side processes. In Figure 4d, the current efficiency exceeds 100% at current densities of 100 and 200 mA·cm^−2^. The current efficiency is close to 100% only for samples anodized at a current density of 15 mA·cm^−2^. It is interesting to note that 100 mA·cm^−2^ is characterized by a significant excess of the current efficiency η*_F_* relative to unity at high additive concentrations above 3.5 g·L^−1^. This is obvious when paying attention to the voltage–time responses shown in Figure 2b. It can be seen that at high concentrations of the additive, anodizing ends radically quickly. Taking into account that, according to the literature, the anodizing of pure aluminum and homogenized AlCu alloy proceeds with an efficiency close to 100% [98,99,100,101], then the observed deviation can be explained only by the existence of a large amount of unprocessed aluminum remaining in the PAOF. The study [51] states that the quantity of residual aluminum, estimated from the micrographs of fractured samples, was less than 1% and at the same time, the highest measured current efficiencies of about 103% can therefore be used to estimate the error of the method. However, studies in [82], carried out using samples from this work (see Section 3.6.3. XRD), showed the presence of residual aluminum amounting to a few percent: from 1.5 to 2.2% at current densities, then decreasing from 250 to 2.5 mA·cm^−2^. The amount of residual aluminum for different anodizing conditions in sulfuric acid is approximately from 0.5 to 2.8%, and the nature of the dependences of the current efficiency and residual aluminum on the current density for different concentrations of sulfuric acid is almost identical. Thus, based on the analysis of literature data and current studies, one can conclude that the excess of the current efficiency relative to unity is due to the presence of residual aluminum, and the data on the current efficiency η*_F_* allowed us to draw indirect conclusions about the approximate amount of residual aluminum in PAOF and its nature, depending on the anodizing conditions. Moreover, it is assumed that in a number of cases, the current efficiency, which is close to 100% as indicated in the literature, is an overestimated value, since the charge spent on side processes can be compensated for by the uncontrolled presence of unoxidized aluminum residues in the PAOF.

### 3.3. Efficiency from dU/dτ

The efficiencies in Figure 4e were obtained by comparing the linear voltage rise dU/dτ with dU/dτ for 100% efficiency. The latter was calculated using literature data for the electric field strength of anodic alumina [102], 8.5 × 10^8^ V·m^−1^, and for the density of alumina [103], 3.0 g·cm^−3^. These values were experimentally confirmed by Páez et al. [99] for alumina barrier film formation in a non-dissolving ammonium pentaborate electrolyte. Efficiencies below 100% can be caused by partial film dissolution or by variation of the electronic oxide conductivity with the anodizing current density, permitting anodic side reactions such as O_2_ evolution or oxidation of bath components. In experiments with complete oxidation of the specimens, the electronic current can be determined from the total electric charge necessary for complete aluminum oxidation (i.e., from the duration of a galvanostatic test). In the case of the anodizing of aluminum in MA without arsenazo-I, it was shown that the electronic current was not higher than 5% [51], which means that the significantly lower efficiency is primarily caused by the loss of Al^3+^ to the electrolyte. In the present work, this kind of analysis of the electronic current was not possible, since the addition of arsenazo-I caused a much stronger scattering of the total anodizing time.

Efficiency diminishes with increasing anodizing current density. This shows that dissolution processes are not merely of a chemical nature. At 15 mA·cm^−2^, the addition of arsenazo-I had little influence on PAOF formation efficiency, and one could observe the same for the case of current efficiency η*_F_*. At current densities of 100 and of 200 mA·cm^−2^, the efficiency diminishes with increasing arsenazo-I concentration. This is, in principle, the expected result for an Al-complexing additive. However, since complexation is also chemical in nature, dissolution by complexation should increase proportional with the time of the test (i.e., efficiency should diminish more at lower applied current densities), so arsenazo-I might have an additional effect on PAOF dissolution, which dominates at lower current density.

Arsenazo-I, as a large molecule, might diminish the potential gradient at the pore ground/electrolyte interface, thus slowing down dissolution at 15 mA·cm^−2^. At higher anodizing current densities, this effect might be eliminated by to the faster movement of the interface, which causes stronger incorporation of arsenazo-I into the PAOF.

### 3.4. Efficiency from Dissolved Aluminum

**Figure 5:**
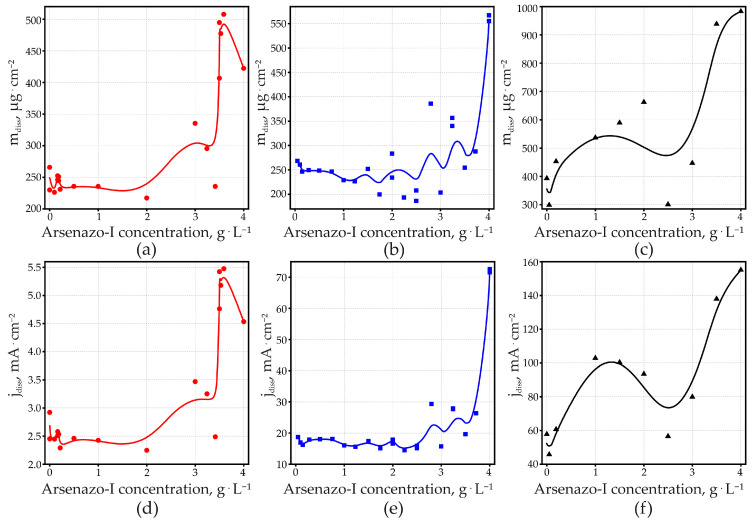
The mass loss per oxide surface area, *m_diss_* (**a**–**c**) for 15, 100, and 200 mA·cm^−2^, respectively, and current density of Al dissolution, obtained from dissolved Al^3+^ (**d**–**f**) for 15, 100, and 200 mA·cm^−2^, respectively, as a function of arsenazo-I concentration for Al in 0.6 M malonic acid.

Analysis of the Al^3+^ content in the anodizing bath after the completion of anodizing gives the dissolution integrated over the whole anodizing time. This essentially describes the behavior during the stationary part, which represents over 95% of the total anodizing time. Figure 5 shows that Al-dissolution increases as the galvanostically applied anodizing current density is increased. This confirms that the diminished efficiency, deduced from dU/dτ, is (at least partially) caused by increased Al dissolution. Efficiencies, obtained from dissolved Al^3+^ at 15 mA·cm^−2^ (Figure 4f) agree with the efficiencies calculated from dU/dτ (Figure 4e) within ±0.1. The discrepancies between the two methods increased for higher current densities and for higher arsenazo-I concentrations. This is most evident for the efficiency at 200 mA·cm^−2^, obtained from Al^3+^ concentration. It is the only curve that shows an increase in efficiency with an increase in the arsenazo-I concentration.

One must keep in mind that the efficiencies in Figure 4e were calculated with a fixed value for electric field strength and density of the PAOF. Therefore, the discrepancy between the two measurement methods obviously demonstrates that the PAOF properties change as a function of the anodizing current density and of the arsenazo-I concentration. It is also worth remembering that the efficiency derived from dU/dτ refers to the dissolution of a barrier layer, where pore formation is at the beginning, whereas the analysis of Al^3+^ in the electrolyte characterizes dissolution through pores, whose length surpasses the thickness of the barrier layer by orders of magnitude.

For 15 and 100 mA·cm^−2^, PAOF dissolution increased for higher arsenazo-I concentrations, according to the expected influence of a complexing additive.

As discussed further on, microscopic examination points to the occurrence of localized breakdown events in the PAOFs formed at 200 mA·cm^−2^ at higher arsenazo-I concentrations. This might be the reason for the changed behavior under these conditions. Since these breakdown events presumably occur only above some critical potential, this change in behavior was not detected when efficiency was determined from the initial voltage rise dU/dτ, as seen in Figure 4e.

A general observation in nearly all tests was that higher arsenazo-I concentrations resulted in a stronger scattering of the experimental results.

### 3.5. Volume Expansion Factor

**Figure 6:**
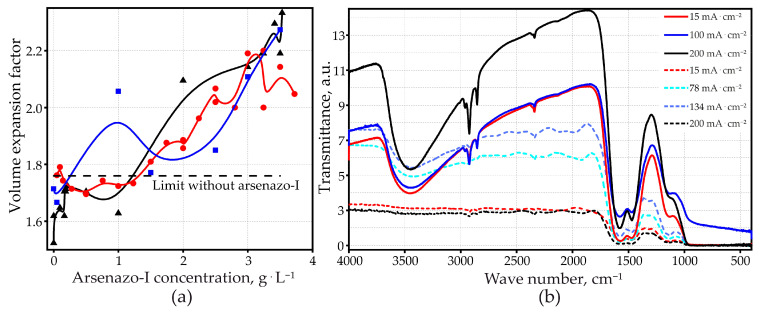
(**a**) Volume expansion factor, as a function of arsenazo-I concentration for Al in 0.6 M malonic acid; FTIR-spectroscopy: malonic acid without arsenazo-I, *j_a_* = 15, 78, 134, 200 mA·cm^−2^ (dashed lines); (**b**) malonic acid with 3 g·L^−1^ arsenazo-I, *j_a_* = 15, 100, 200 mA·cm^−2^ (solid lines).

The volume expansion factor increased with increasing arsenazo-I concentration, up to about 2.3 for the solutions with the highest arsenazo-I concentrations used in this work (Figure 6a). The behavior was similar for the three examined current densities. There is a clear difference between the present results and the results obtained recently without the addition of arsenazo-I [51]. In pure 0.6 M MA, the volume expansion factor did not exceed a limit of 1.76. At a current density higher than approximately 40 mA·cm^−2^, it was observed that the volume expansion factor remained at this limit, independent of the applied anodizing current density. This was attributed to a mechanism of self-adjustment of the active surface area where the applied current passed through. This allowed the system to keep the growth conditions constant, thus always leading to the same PAOF thickness, independent of the applied current density. A consequence of this self-adjusting growth mechanism was a higher degree of PAOF ordering [51].

The results obtained in arsenazo-I containing the electrolyte indicate that the self-adjusting mechanism no longer worked when the arsenazo-I concentration reached some 1.5 g·L^−1^. The volume expansion factor then surpassed the limit of 1.76 found in pure MA.

As described below, the morphology of the PAOFs grown under these conditions was disordered, thus confirming that the self-adjusting mechanism was suppressed by the addition of arsenazo-I.

### 3.6. Film Structure

#### 3.6.1. Fourier Transform Infrared Spectroscopy

The FTIR spectra of completely anodized samples showed significant differences in the OH-stretching region around 3500 cm^−1^ (Figure 6b). The absorption peak was weak in MA at the low applied current density and increased with an increase in applied current density. Absorption by OH-groups also increased when 3 g·L^−1^ arsenazo-I was added to the electrolyte. The incorporation of hydroxyl groups into the PAOF means an increase in structural disorder. Consequently, an oxide layer of lower density is formed. This is in agreement with Figure 6a, which shows that the volume expansion factor increases with an increase in arsenazo-I concentration. According to this interpretation of the FTIR spectra, a higher volume expansion factor should also be expected for higher applied current densities. Indeed, this behavior has been observed in an arsenazo-I-free electrolyte and published recently [51]. With the addition of arsenazo-I, however, the results are more scattered and the influence of the current density becomes, therefore, less clear (Figure 6b).

#### 3.6.2. X-ray Photoelectron Spectroscopy

**Figure 7:**
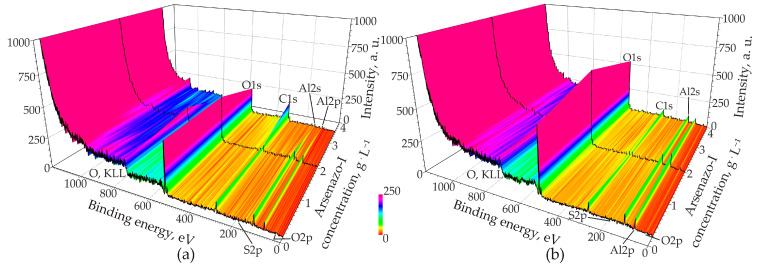
Survey XPS spectra of porous aluminum oxide film samples formed at various concentrations of arsenazo-I at current densities of (**a**) 15 and (**b**) 200 mA·cm^−2^ after 40 min of sputtering.

XPS spectra of anodized specimens were measured after 40 min of sputtering. Figure 7 shows an overview of the spectra of samples formed at 15 and 200 mA·cm^−2^ at three different concentrations of arsenazo-I.

**Figure 8:**
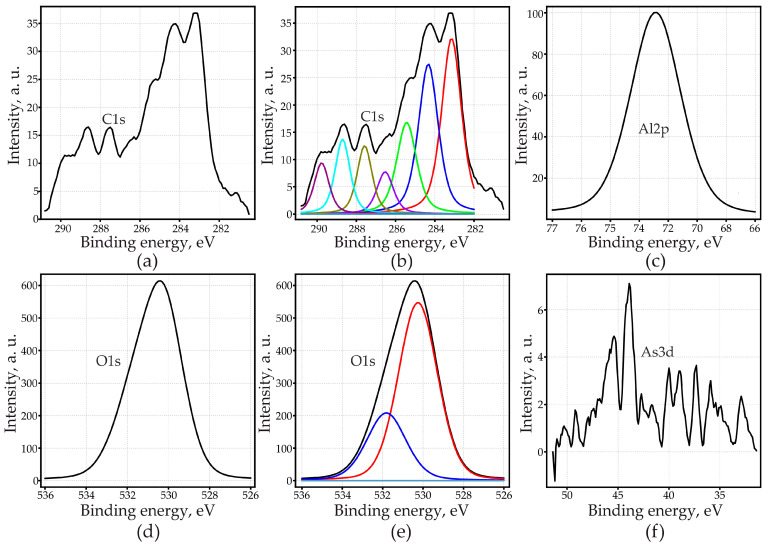
XPS-peaks of porous aluminum oxide films, measured after 40 min of sputtering (*j_a_* = 200 mA·cm^−2^; 2.5 g·L^−1^ arsenazo-I): (**a**) C1s; (**b**) deconvolution of C1s-peaks; (**c**) Al2p; (**d**) O1s; (**e**) deconvolution of O1s-peak; (**f**) As3d.

**Figure 9:**
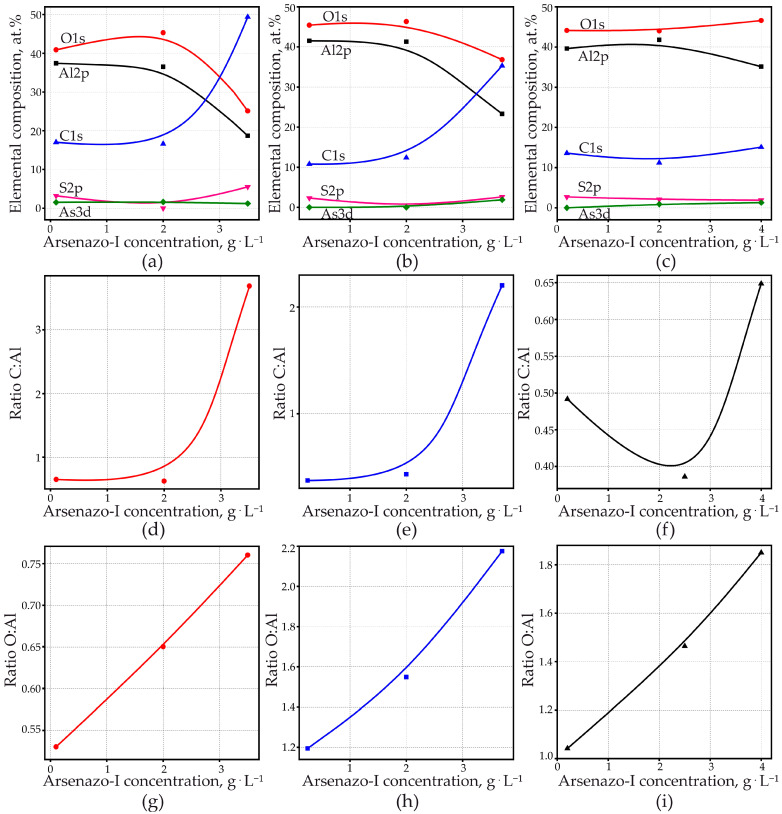
Elemental composition of porous aluminum oxide films according to XPS spectra (**a**–**c**); analysis of XPS spectra anodized samples, after 40 min of sputtering: (**d**–**f**) relation of peak areas C1s/Al2p; (**g**–**i**) atomic relation of O/Al. Galvanostatic anodizing with 15 (**a**,**d**,**g**), 100 (**b**,**e**,**h**), and 200 mA·cm^−2^ (**c**,**f**,**i**), respectively.

These spectra can clearly identify oxygen, aluminum, carbon, and small amounts of arsenic and sulfur. It can be seen from Figure 7 that, depending on the anodizing conditions, the ratio of the elements that make up PAOF changes significantly. This observation prompted a more thorough study of the elemental composition of PAOF. The XPS of all tested samples after 40 min of sputtering are shown in Figures S1–S9 of the Supplementary Materials. Figure 8 shows an example of the peaks obtained for O, Al, C, and As, and Figure 9a–c shows the results of quantitative elemental analysis of PAOF depending on the concentration of the arsenazo-I additive, formed at different anodic current densities obtained from the ratio of peak areas and its respective sensitivity factors. Charge corrections were made by defining the position of the C1s (C–C/C–H) peak as 284.8 eV. The C1s spectrum proved to be complex due to the variety of different binding conditions of carbon in MA and in the arsenazo-I molecule and its derivates. C1s peaks could be expected between the C–C/C–H–peak at 284.8 eV and the O=C–O–peak, which should appear at higher values about 4.5 eV, according to the literature [104].

The measured C1s spectra (Figure 8a) extended over an energy range of at least 6.5 eV. Most of the spectra could be fitted with three higher peaks with 1.2 eV FWHM at the lower energy side and four smaller peaks with 1.0 eV FWHM at the higher energy side, as shown in the example in Figure 8b. The smaller peaks outside the expected energy range were probably caused by plasmon losses of aromatic carbon in arsenazo-I. It was not possible, without additional measurements, to unambiguously separate plasmon loss, arsenazo-I, and the MA peaks. Therefore, the relation of the peak areas C1s/Al2p as a relative measurement of organic components incorporated into the PAOF (Figure 9d–f) was used. For the C1s peaks, an energy range of 10 eV was used for analysis, thus including all observed XPS peaks as well as peaks due to other interactions. The analysis can, therefore, not be considered as quantitative. It can be seen (Figure 9d–f) that, for the three examined anodizing current densities, the content of organic compounds in the PAOF increased with increasing arsenazo-I concentration in the electrolyte.

O1s appeared as a broad peak with FWHM 3.0 eV (Figure 8d,e). The peak should be composed of Al–O–Al (i.e., signals from bindings of O within the amorphous Al_2_O_3_ structure and from O bound to organic compounds as well as O belonging to hydroxyl groups). According to the literature, the energy of the signal from the strongly ionic binding in Al_2_O_3_ should be approximately 0.7 eV lower than the one from the bindings that involve C or H [105]. The energy difference is obviously small compared with the broadness of the O1s peak, thus turning the separation of the peak into the different electronic states into a rather arbitrary procedure. The Al2p peak also did not allow us to differentiate bindings (Figure 8c). Figure 9g–i shows the atomic ratio of O/Al, obtained from the ratio of peak areas O1s/Al2p and the respective sensitivity factors. An increase in the atomic ratio O/Al was observed with increasing arsenazo-I concentration for the three anodizing current densities. The O/Al ratio also increased with increasing current density.

The As peak was visible in the spectra (Figure 8f), however, it was very close to the detection limit. Therefore, a tendency of the As3d content could not unequivocally be determined from a comparison of the peaks. However, one can capture several observations.

First, at a minimum current density of 15 mA·cm^−2^, the arsenic concentration in the PAOF was practically independent of the arsenazo-I concentration in the electrolyte and was approximately 1.2–1.6 at. %. An increase in the concentration of the complexing additive led to an increase in the S content, which is also a part of arsenazo-I, and a sharp increase in the C content and a decrease in aluminum and oxygen. An increase in the arsenazo-I concentration led to the fact that the content of the impurity element, carbon, was higher than that of the main elements that make up PAOF, Al and O, reaching 49 at. % (Figure 9d) and the sulfur concentration reached 5.5 at. %. Such a high concentration of impurity elements was not achieved in any of our described experiments and at one time served as the basis for the following brief communication [95].

Second, an increase in the current density is guaranteed to lead to a decrease in the content of carbon and sulfur in PAOF (Figure 9b,c,e,f), although an increase in the concentration of arsenazo-I will still noticeably increase the concentration of carbon at a current density of 100 mA·cm^−2^, and, which very curiously, had practically no effect on the carbon content at 200 mA·cm^−2^. In this case, at both high values of the anodic current densities (100 and 200 mA·cm^−2^), an increase in the concentration of the arsenic-containing additive led to an increase in the concentration of arsenic in PAOF from zero to 1.9 and 1.3 at. %, respectively. In this case, the sulfur content was approximately half less than that in PAOF formed at 15 mA·cm^−2^ at any concentration of arsenazo-I. That is, at a high current density (200 mA·cm^−2^), only at high concentrations of arsenazo-I is arsenic incorporated into the PAOF composition in detectable amounts, but this is not accompanied by an increase in the carbon concentration in the PAOF. The latter observation is especially interesting in combination with the detection of an inorganic arsenic compound in PAOF (see Section 3.6.3. X-ray Diffractometry).

#### 3.6.3. X-ray Diffractometry

**Figure 10:**
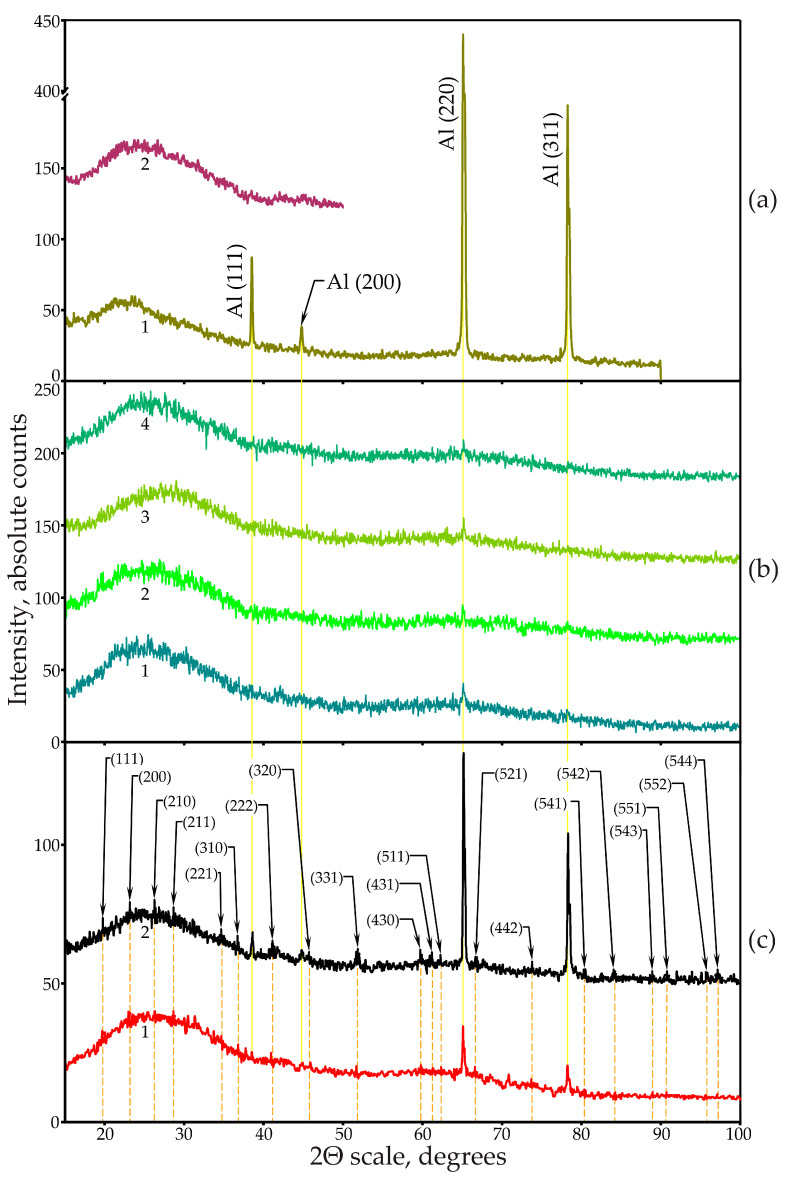
(**a**) XRD spectra of the initial aluminum foil (curve 2) and a glass substrate with an adhesive layer applied to foil (curve 1). (**b**) XRD spectra of porous aluminum oxide film samples obtained in 0.6 M malonic acid without additive at current densities 2.54, 60.7, 150 and 243 mA·cm^−2^ (curves 1, 2, 3 and 4 respectively). (**c**) XRD spectra of porous aluminum oxide film samples obtained in 0.6 M malonic acid with an addition of arsenazo-I 3.24 g·L^−1^ and a current density of 15 mA·cm^−2^ (curve 1), and 2.50 g·L^−1^ and a current density of 200 mA·cm^−2^ (curve 2).

XRD studies of some PAOF samples formed in both 0.6 M pure MA and with the addition of arsenazo-I as well as reference samples, a glass substrate, and initial aluminum foil were carried out. Figure 10a shows the XRD spectrum of the initial aluminum foil glued to a glass substrate to ensure mechanical stability (curve 1) and a fragment of the diffraction pattern of a glass substrate with an adhesive layer applied to it (curve 2). Figure 10b shows the XRD patterns of PAOF formed in a pure 0.6 M MA solution without a complexing additive at current densities of 2.54, 60.7, 150, and 243 mA·cm^−2^ (curves 1, 2, 3, and 4, respectively). Figure 10c shows the XRD patterns of the samples formed in the MA with the addition of arsenazo-I. Curve 1 corresponds to a sample formed at a current density of 15 mA·cm^−2^ and an arsenazo-I concentration of 3.24 g·L^−1^, and curve 2 to a sample formed at a current density of 200 mA·cm^−2^ and additive concentration of 2.50 g·L^−1^. XRD confirmed the amorphous character of all studied PAOFs. Aluminum peaks were identified in all XRD spectra of PAOF (indicated on diffractogram 1 in Figure 10a).

The PAOFs that formed in the electrolyte with arsenazo-I, however, showed small peaks that could be identified as Na_1,5_Al_2_(OH)_4,5_(AsO_4_)_3_·7H_2_O (the peaks are indicated in the figure, XRD spectrum 2) with a cubic cell with a lattice constant of 7.718 Å (according to our calculations) or 7.715 Å (according to the International Center for Diffraction Data [106], file no. 30-1145).

When comparing the XRD patterns of PAOF formed at 15 and 200 mA·cm^−2^, shown in Figure 10c, curves 1 and 2, respectively, it should be noted that the peaks of the detected inorganic arsenic compound, although of a rather low intensity, were still quite clearly visible in the XRD pattern of a sample formed at 200 mA·cm^−2^ and only practically in some cases were barely guessed on the XRD pattern of a sample obtained at a current density of 15 mA·cm^−2^. This is especially impressive considering that at 15 mA·cm^−2^, anodization took place in an electrolyte with a higher arsenazo-I concentration than at 200 mA·cm^−2^. This indicates that the compound synthesis efficiency with the formula Na_1,5_Al_2_(OH)_4,5_(AsO_4_)_3_·7H_2_O increases not only with an increase in the content of the arsenic-containing additive in the electrolyte, but above all, with an increase in the current density. This consideration is in full agreement with the observations outlined in Section 3.6.2. X-ray Photoelectron Spectroscopy.

Thus, based on the data on the absolute amount of the main (Al, O) and impurity (S, As) elements as well as on their ratio depending on the anodizing conditions, obtained as a result of XPS analyses based on the data on the identification of the arsenic compound with formula Na_1,5_Al_2_(OH)_4,5_(AsO_4_)_3_·7H_2_O in the composition of PAOF and the dependence of its amount on the anode current density obtained by XRD studies, the authors can conclude that the low current density promotes the incorporation of arsenic into the structure of PAOF in the composition of the arsenazo-I molecule, and an increase in the density of the anodic current leads to its destruction, the release of arsenic in the form of an acidic residue of arsenic acid, and the formation of the corresponding compound of the indicated composition, which is also incorporated into the structure of PAOF.

**Figure 11:**
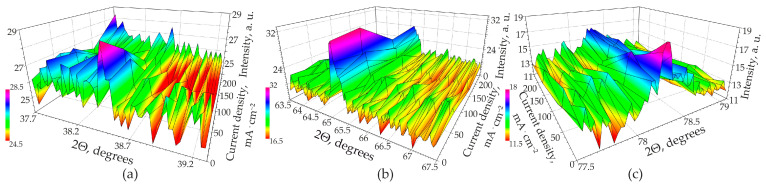
Dependence the XRD peak intensity of aluminum on the anodic current density during galvanostatic anodizing in pure 0.6 M MA in the 2Θ ranges of (**a**) 37.7–39.1°, (**b**) 63.5–67.5°, and (**c**) 77.5–79.1°.

To identify residual aluminum in the PAOF, formed in MA solution without the additive more accurately, three peaks with maxima at 38.51, 65.11, and 78.27° were selected. Figure 11a–c shows the dependence of the intensity of the mentioned peaks on the density of the anodic current in the 2Θ ranges of 37.7–39.1°, 63.5–67.5°, and 77.5–79.1°, respectively (Figure 11a–c). Examination of Figure 11a–c at a qualitative level clearly shows a decrease in the intensity of the peaks with an increase in the current density. To confirm this conclusion, a quantitative assessment of the residual aluminum content in PAOF formed in 0.6 M MA containing no arsenazo-I additives was performed.

**Figure 12:**
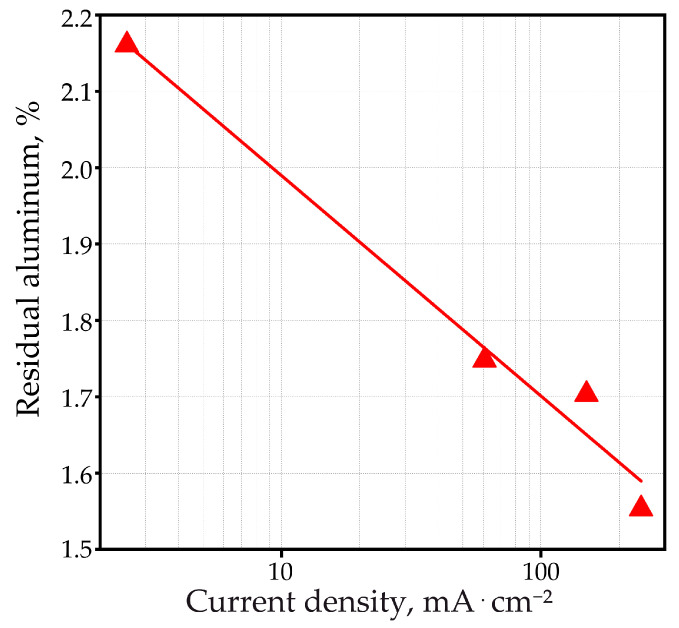
Quantification of residual aluminum content versus anodic current density based on areas under the 65.11° aluminum peak during galvanostatic anodizing in pure 0.6 M malonic acid.

The method for quantifying residual aluminum is described in [82]. For quantitative assessment, the area of the most intense peak of aluminum at 65.11° was considered, and the % proportion of unreacted (residual) aluminum *R*_Al_ was calculated using the formula:

(11) $$R_{\mathrm{Al}}=\frac{S_{\mathrm{Al},PAOF}}{S_{\mathrm{Al},Me}}\cdot 100\%$$

where *S*_Al,*PAOF*_ and *S*_Al,*Me*_ are the areas under the 65.11° aluminum peak for the PAOF and the original foil, respectively. The results are plotted in Figure 12. The associated uncertainty was estimated to be ±4%. The results of calculating the amount of residual aluminum are shown in Figure 12. The content of non-anodized aluminum decreases according to the law of logarithms with an increase in the current density of anodizing in accordance with the equation that describes the considered dependence well enough (R-Square (COD) is 0.97848).

(12) $$R_{\mathrm{Al}}=2.27838-0.28868\cdot \mathrm{lg}\left(j_{a}\right)$$

and is, according to new data, approximately from 1.5 to 2.2% for current densities of 250 and 2.5 mA·cm^−2^, respectively. These results contradict the information given earlier in [51], which states that the quantity of residual aluminum, estimated from the micrographs of fractured samples, is less than 1%. It seems that the data refined in this work as a result of XRD studies are more reliable. The X-ray spot of the used device has a sufficiently large area (on the order of 1 cm^2^ or more, depending on the angle of incidence of the X-ray beam), which allows the signal over a large region of the total sample to be averaged, and with the help of SEM or TEM, one can see several (a small number only) random points.

At the same time, as can be seen in Figure 10c, an increase in the current density from 15 to 200 mA·cm^−2^ upon anodizing aluminum in MA with arsenazo-I quite obviously led to a significant increase in the amount of non-anodized aluminum. This is expressed in an increase in the relative height of the 65.11° peak of aluminum from 20 to 75 counts, and the peak corresponding to 78.27° from 10 to 50 counts. This tendency, as one can see, is in contrast to that found for the case of anodizing in a solution of pure MA and nevertheless, was unambiguously confirmed by the data on the current efficiency η*_F_* (shown in Figure 4d), and is consistent with the considerations stated above in Section 3.2. Current Efficiency.

### 3.7. Film Morphology

**Figure 13:**
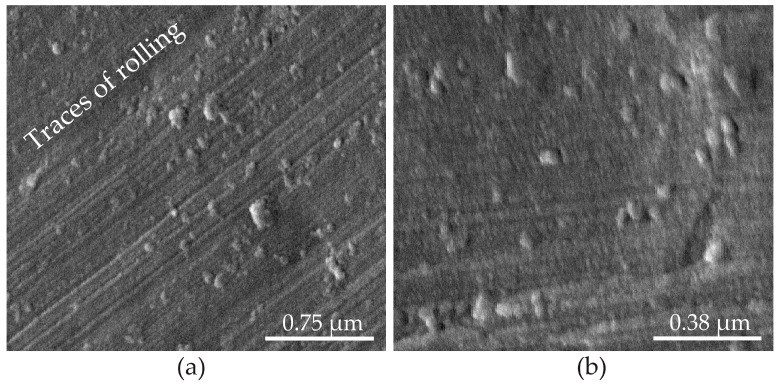
SEM images of the original aluminum foil surface at various resolutions: (**a**) ×40,000 and (**b**) ×80,000.

Figure 13 shows the SEM images of the initial aluminum foil surface at different resolutions. On the surface, irregularly located rounded bulges and parallel stripes are clearly visible, representing traces of rolling.

**Figure 14:**
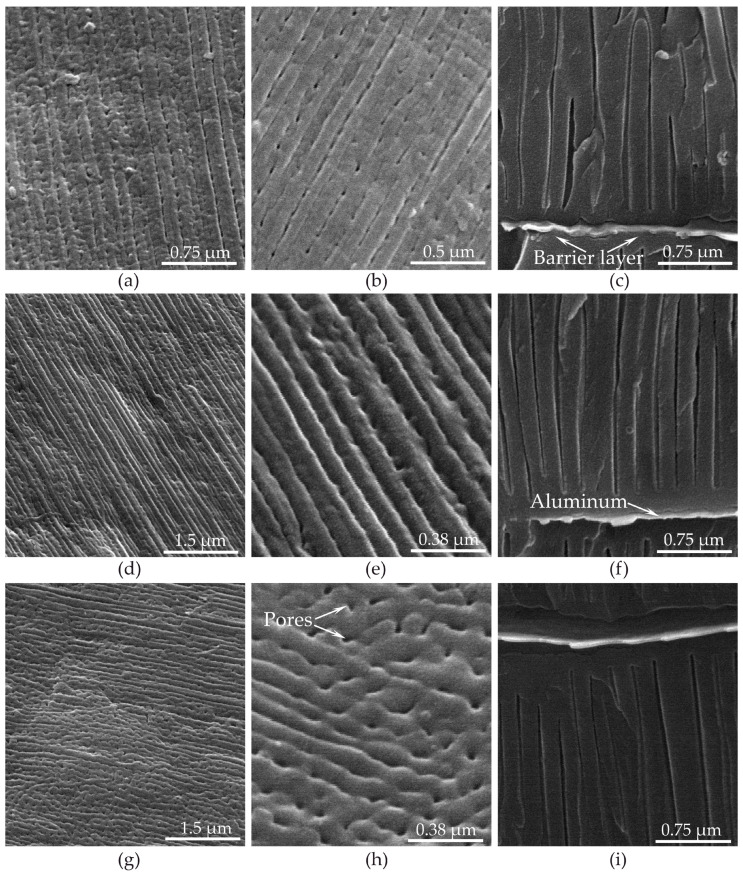
SEM images at various resolutions of porous aluminum oxide film surfaces (**a**,**b**) and cross-section, (**c**) formed in the galvanostatic mode in 0.6 M malonic acid without arsenazo-I at an anodic current density of 6.0 mA·cm^−2^, porous aluminum oxide film surfaces (**d**,**e**), and cross-section (**f**) formed at an anodic current density of 50 mA·cm^−2^. Porous aluminum oxide film surfaces (**g**,**h**) and cross-section (**i**) formed at anodic current density of 500 mA·cm^−2^.

Figure 14 shows the SEM of the surface (a, b, d, e, g, h) and cross-section (c, f, i) of PAOFs formed at three values of the anodic current densities differing by an order of magnitude: 6, 50, and 500 mA·cm^−2^ (a–c, d–f and g–i, respectively).

An increase in the anodic current density led to a decrease in surface etching in the direction of the rolled tracks, where the PAOF surface became more even and defect-free. A layer of non-anodized aluminum was clearly visible on each of the cross-sections.

**Figure 15:**
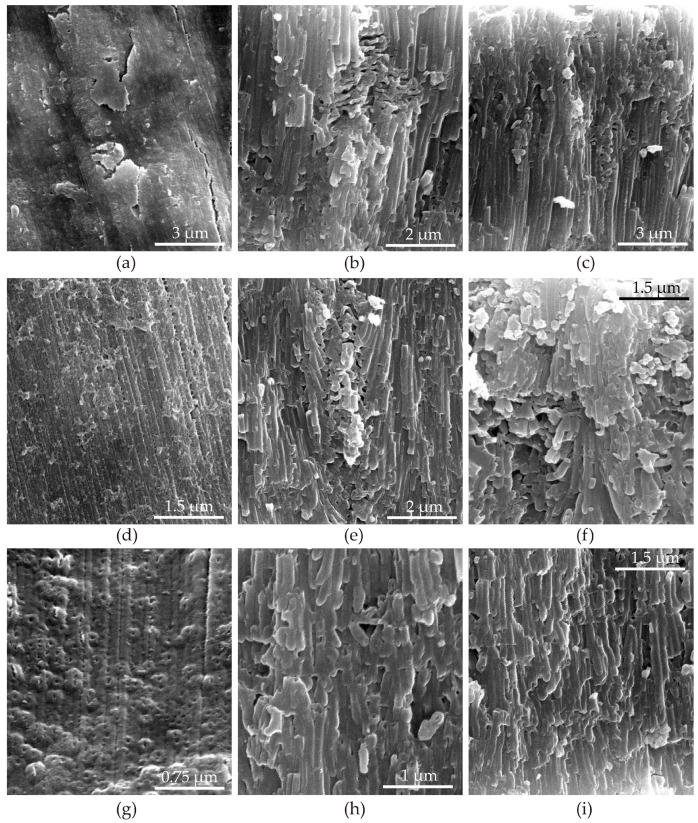
SEM images at various resolutions of porous aluminum oxide film surface (**a**) and cross-sections (**b**,**c**) formed in galvanostatic mode in 0.6 M malonic acid at an anodic current density of 15 mA·cm^−2^ with 3.42 g·L^−1^ arsenazo-I additive, porous aluminum oxide film surface (**d**) and cross-sections (**e**,**f**) formed at an anodic current density of 100 mA·cm^−2^ with 3.72 g·L^−1^ arsenazo-I additive, and porous aluminum oxide film surface (**g**) and cross-sections (**h**,**i**) formed at an anodic current density of 200 mA·cm^−2^ with 4.0 g·L^−1^ arsenazo-I additive.

Figure 15 and Figures S10–S20 in the Supplementary Materials show the morphology of PAOFs in cross section as a function of anodizing current density and of arsenazo-I concentration. It can be observed that these two parameters exert opposite effects on PAOF morphology: increasing current density led to increased ordering of the PAOF. This agrees with the finding made recently in MA without arsenazo-I [51] and in the Figure 14 images. In this case, the increase in ordering was attributed to a growth mechanism with a self-adjusting active area (burning spot), which is probably also the reason for the current independent volume expansion factor observed under these conditions. This mechanism is obviously suppressed by the addition of arsenazo-I. The influence of arsenazo-I is visible by the change in the volume expansion factor (Figure 6a) and by the growing morphological disorder of the PAOF (Figure 15b,c,e,f,h,i). One can see pores that deviate considerably from the growth direction perpendicular to the surface. This locally occurring collective deviation creates macroscopic defects. In the whole PAOF, pores were found that did not extend until the final position of the metal–oxide interface (i.e., pore growth stopped locally, thus causing the branching of other tunnels). The reason for this disordering effect of arsenazo-I might be found in the lowering of the electric field as the voltage–time (Figure 2) or voltage–applied charge (Figure 3) transients have shown that arsenazo-I considerably lowers the stationary anodizing voltage. Other researchers have already confirmed that a high electric field is beneficial for self-ordering in PAOF [107,108]. Moreover, the detection of traces of a crystalline arsenic-containing phase demonstrates the strong influence of arsenazo-I on the structure of the PAOF. Therefore, PAOF growth becomes more disordered than in pure MA. Microscopic examination of the anodized surfaces (Figure 15a,d,g) shows that, beside the rolling lines, at low magnification, featureless surfaces combined with macroscopic cracks at 15 and 100 mA·cm^−2^, while at 200 mA·cm^−2^, cracks practically disappeared. Cracking can be a result of both micro-breakdowns and the result of PAOF cracking due to mechanical stresses arising from the incorporation of an extraordinary amount of impurity ions (see Section 3.6.2. X-ray Photoelectron Spectroscopy, Figure 9a,b,d,e). It is curious to note that with an increase in the current density, the nature of the surface changed: the number of macroscopic cracks decreased, but halos were observed, sub-micrometer protuberances, obviously with a central discharge channel, whose number increased with increasing arsenazo-I concentration. This points to the appearance of dielectric PAOF breakdown.

The morphology is similar to the one observed in the case of dielectric breakdown of barrier layers, for example, sparking of PAOFs on Mg-alloys [109]. The unusually strong oscillations of the anodic voltage corroborate this interpretation.

Another interpretation of the formations found on the PAOF surface at a current density of 200 mA·cm^−2^ is also possible. The formations can be viewed as pore holes located at the tops of the projections. Then, the second possible reason may be the deposits around the pore holes of chemical compounds formed with the participation of aluminum ions, carried out from the bottom of the pores from the dissolving oxide as it forms and dissolves. With this consideration, it can be assumed that these protrusions are formed by the compound Na_1,5_Al_2_(OH)_4,5_(AsO_4_)_3_·7H_2_O, which is most likely formed near the pore holes as a result of the interaction of aluminum ions and the arsenazo-I electrolysis products. The increase in the number of such protrusions with increasing current density and arsenazo-I concentration (see Supplementary Materials, Figures S16–S19) is in good agreement with the results of the XRD investigations (see Section 3.6.3. X-ray Diffractometry and Figure 10c) and does not contradict any of the possible explanations.

The presence of micro-breakdowns at both high and low values of the anodic current densities was confirmed by the oscillating nature of the voltage–time (Figure 2) or voltage–applied charge (Figure 3) dependences.

## 4. Conclusions

In the present work, galvanostatic aluminum anodizing at 0.6 M malonic acid (MA) containing up to 4.0 g·L^−1^ arsenazo-I was carried out; the composition, structure, and morphology of the obtained porous aluminum oxide films (PAOFs) were studied. The studies performed allowed us to draw the following conclusions:

1. The addition of arsenazo-I to MA has considerable influence on the anodizing behavior of pure aluminum. In detail, this means an influence on the dissolution current density and, consequently, on the efficiency of PAOF formation, conductivity, the volume expansion factor, composition, structure, and morphology. Generally, the influence of arsenazo-I increased with increasing concentration and with increasing current density.
2. The data on the current efficiency η*_F_* allowed us to make a conclusion on the amount of residual aluminum in the PAOF. A significant excess in some cases of the current efficiency η*_F_* relative to unity is due to the presence of a large number of islands of non-anodized aluminum.
3. Acceleration of metal dissolution with increasing arsenazo concentration agrees with the complexation of Al^3+^ by arsenazo-I. However, the observed stronger influence at higher current densities reveals the superposition of other effects caused by arsenazo-I.
4. FTIR-spectra and XPS measurements indicate lower coordinated aluminum and thus less dense oxide due to the increasing number of hydroxyl groups with increasing arsenazo-I concentration as well as an increasing quantity of carbon components in the PAOF. As a consequence, the volume expansion factor increases with increasing arsenazo-I concentration.
5. The higher number of defects caused by these structural changes leads to a higher ionic conductivity of the PAOF, which results in a reduced anodizing voltage when compared with anodizing in an arsenazo-free solution.
6. XPS and XRD measurements indicate that at a low anodizing current density (15 mA·cm^−2^), the incorporation of arsenic occurs, presumably, in the composition of complex compounds of arsenazo-I with aluminum, and at a high current density due to the formation of an inorganic compound with the formula Na_1,5_Al_2_(OH)_4,5_(AsO_4_)_3_·7H_2_O, and the arsenic for the formation of this compound was formed as a result of the destruction of arsenazo-I molecules at a high anodic current density (200 mA·cm^−2^).
7. The reduced anodizing voltage and, consequently, the reduced electric field is most likely the reason for the absence of the self-adjusting mechanism, a PAOF formation mechanism observed in pure MA, where the self-adjustment of the active surface area leads to PAOF formation conditions that remain constant over a certain range of applied current densities. An increased degree of self-ordering and a current-independent volume expansion factor were morphological characteristics, observed under these growth conditions. The addition of arsenazo-I leads to the loss of these properties: PAOFs are less ordered and the growth factor changes as a function of anodizing current density, indicating that the self-adjusting mechanism is no longer operative. This agrees with former observations that higher electric fields favor self-ordering of the PAOF [107,108].
8. The incorporation of organic components from the electrolyte might also lower the dielectric constant of the PAOF. It is generally accepted that a lower dielectric constant means a higher dielectric strength (i.e., a higher breakdown voltage). This also agrees with the absence of the self-adjusting mechanism, which represents a kind of non-destructive breakdown mechanism. However, other dielectric breakdown events, destructive and more similar with sparking in barrier films, were observed. Self-ordering of the PAOF is totally lost under these conditions.

The results of this study show the possibilities for introducing anomalous amounts of impurity elements into the composition of the PAOF in order to create materials with a complex of unusual properties in the future.

## Data Availability

Not applicable.

## Supplementary Materials

The following are available online at www.mdpi.com/1996-1944/14/17/5118/s1, Figures S1–S3: X-ray photoelectron spectroscopy analysis results of the porous aluminum oxide films obtained in 0.6 M malonic acid at 15 mA·cm^−2^ current density and 9.76·10^−2^, 2.0, and 3.5 g·L^−1^ arsenazo-I addition, Figures S4–S6: X-ray photoelectron spectroscopy analysis results of the porous aluminum oxide films obtained in 0.6 M malonic acid at 100 mA·cm^−2^ current density and 2.79·10^−1^, 2.0, and 3.72 g·L^−1^ arsenazo-I addition, and Figures S7–S9: X-ray photoelectron spectroscopy analysis results of the porous aluminum oxide films obtained in 0.6 M malonic acid at 200 mA·cm^−2^ current density and 1.90·10^−1^, 2.0, and 4.0 g·L^−1^ arsenazo-I addition; Figures S10–S13: Scanning electron microscopy images of porous aluminum oxide film surfaces and cross-sections obtained in 0.6 M malonic acid at 15,0 mA·cm^−2^ current density, and 3.415 and 4.0 g·L^−1^ arsenazo-I addition, Figures S14–S16: Scanning electron microscopy images of porous aluminum oxide film surfaces and cross-sections obtained in 0.6 M malonic acid at 100 mA·cm^−2^ current density and 3.0 and 3.721 g·L^−1^ arsenazo-I addition, Figures S17–S20: Scanning electron microscopy images of porous aluminum oxide film surfaces and cross-sections obtained in 0.6 M malonic acid at 200 mA·cm^−2^ current density and 2.0 and 4.0 g·L^−1^ arsenazo-I addition.
