# Supplementary material for: Peculiar Porous Aluminum Oxide Films Produced via Electrochemical Anodizing in Malonic Acid Solution with Arsenazo-I Additive
Source: Materials (Basel). 2021 Sep 6;14(17):5118. doi: 10.3390/ma14175118 (PMC8433957; doi:10.3390/ma14175118)
Supplement: Supplementary file 1 [file materials-14-05118-s001.zip › materials-1333416-supplementary.pdf]

materials

MDPI

Supplementary Materials

Peculiar Porous Aluminum Oxide Films Produced via Electrochemical Anodizing in Malonic Acid Solution with Arsenazo-I Additive

Alexander Poznyak [1,2], Gerhard Knörnschild [3], Anatoly Karoza [4], Małgorzata Norek [5] and Andrei Pligovka [2,6,*]

[1] Department of Electronic Technology and Engineering, Belarusian State University of Informatics and Radioelectronics, 6 Brovki Str., 220013 Minsk, Belarus; poznyak@bsuir.by
[2] Research and Development Laboratory 4.10 "Nanotechnologies", Belarusian State University of Informatics and Radioelectronics, 6 Brovki Str., 220013 Minsk, Belarus
[3] Department of Metallurgy, Federal University of Rio Grande do Sul, Porto Alegre 90040-060, Brazil; Gerhard.Hans@ufrgs.br
[4] Centre for Analytical and Spectral Measurements, B.I. Stepanov Institute of Physics of the National Academy of Sciences of Belarus, 68-2, Nezavisimosti Ave., 220072 Minsk, Belarus; a.karoza@ifanbel.bas-net.by
[5] Institute of Materials Science and Engineering, Faculty of Advanced Technologies and Chemistry, Military University of Technology, 2 Kaliskiego Str., 00-908 Warsaw, Poland; malgorzata.norek@wat.edu.pl
[6] Department of Micro- and Nanoelectronics, Belarusian State University of Informatics and Radioelectronics, 6 Brovki Str., 220013 Minsk, Belarus
* Correspondence: pligovka@bsuir.by; Tel.: +375-44-730-95-81; Fax: +375-17-293-23-56

Citation: Poznyak, A.; Knörnschild, G.; Karoza, A.; Norek, M.; Pligovka, A. Peculiar Porous Aluminum Oxide Films Produced via Electrochemical Anodizing in Malonic Acid Solution with Arsenazo—I Additive. *Materials* **2021**, *14*, 5118. https://doi.org/10.3390/ma14175118

Academic Editor: Alina Pruna

Received: 24 July 2021
Accepted: 31 August 2021
Published: 6 September 2021

Publisher's Note: MDPI stays neutral with regard to jurisdictional claims in published maps and institutional affiliations.

Copyright: © 2021 by the authors. Licensee MDPI, Basel, Switzerland. This article is an open access article distributed under the terms and conditions of the Creative Commons Attribution (CC BY) license (http://creativecommons.org/licenses/by/4.0/).

S1. Porous Aluminum Oxide Film X-Ray Photoelectron Spectra

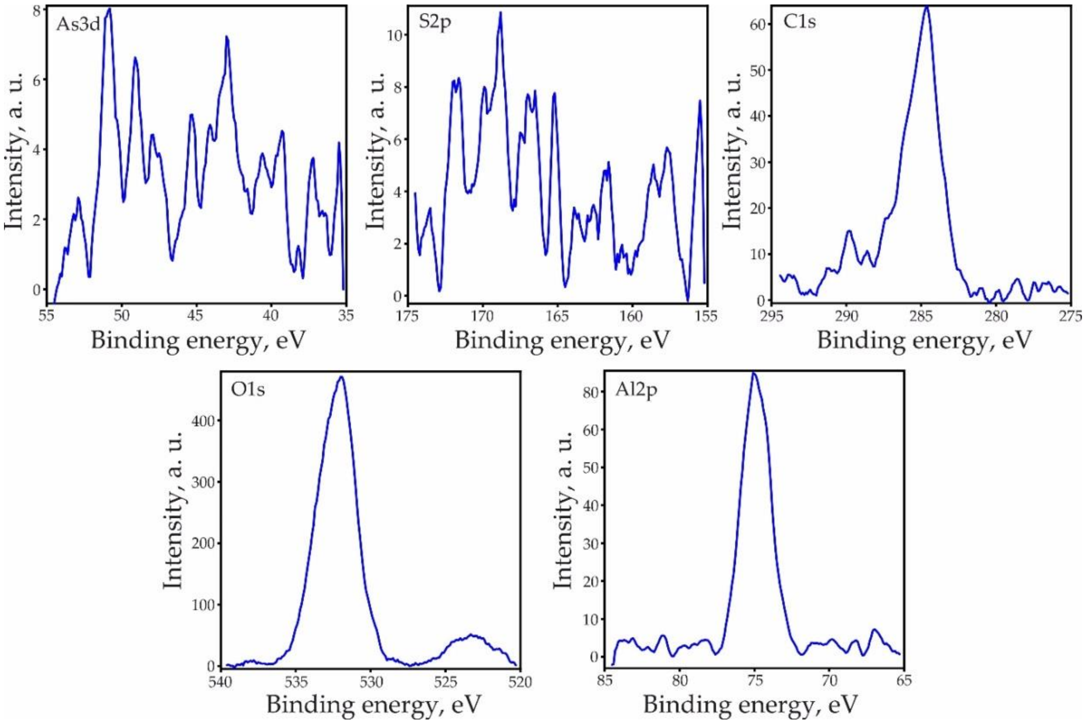

Figure S1. X-ray photoelectron spectra of porous aluminum oxide film formed in 0.6 M malonic acid solution at 15.0 mA·cm^{-2} current density with 9.76·10^{-2} g·L^{-1} arsenazo-I addition.

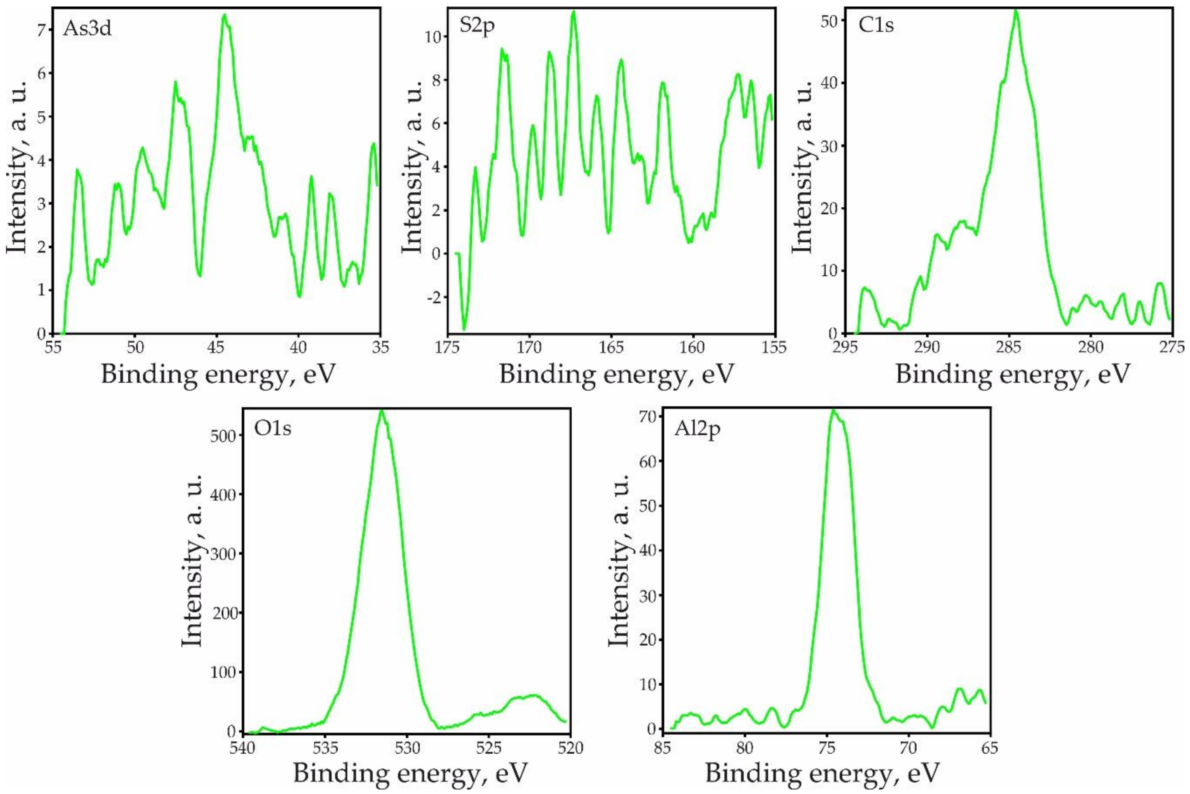

Figure S2. X-ray photoelectron spectra of porous aluminum oxide film formed in 0.6 M malonic acid solution at 15.0 mA·cm^{-2} current density with 2.0 g·L^{-1} arsenazo-I addition.

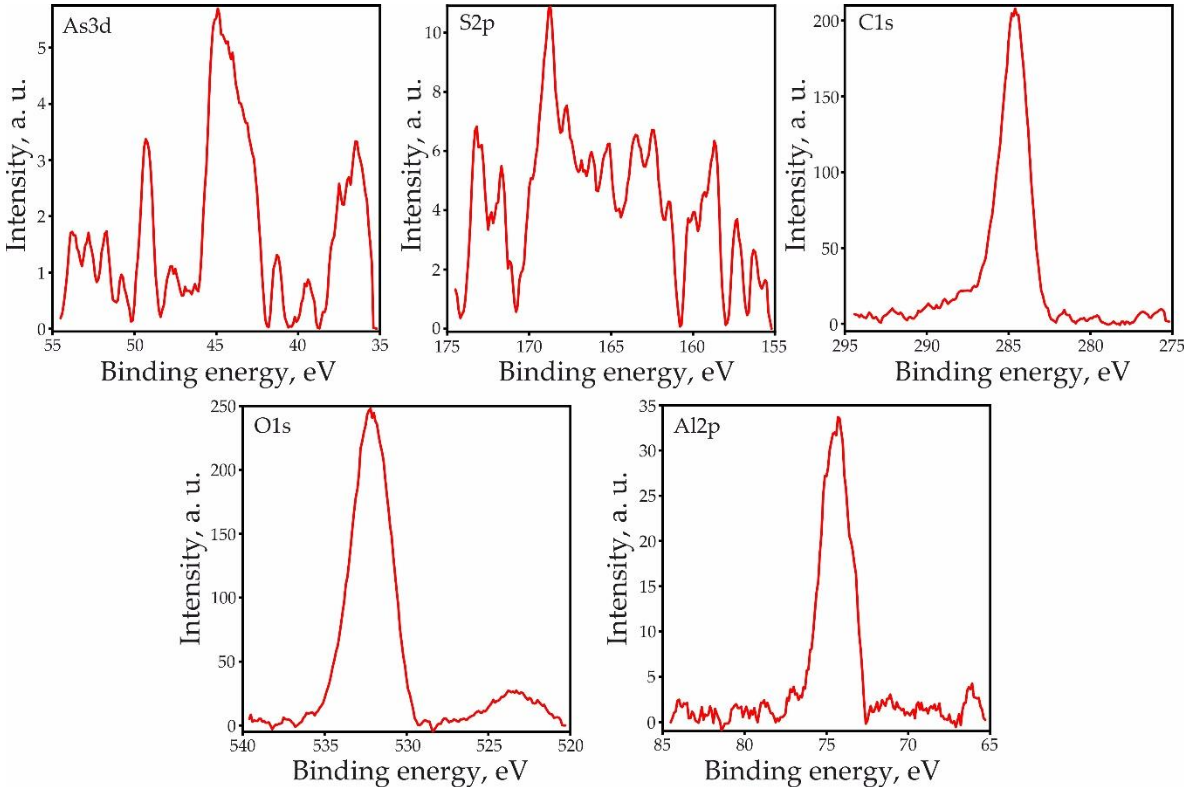

Figure S3. X-ray photoelectron spectra of porous aluminum oxide film formed in 0.6 M malonic acid solution at 15.0 mA·cm^{-2} current density with 3.5 g·L^{-1} arsenazo-I addition.

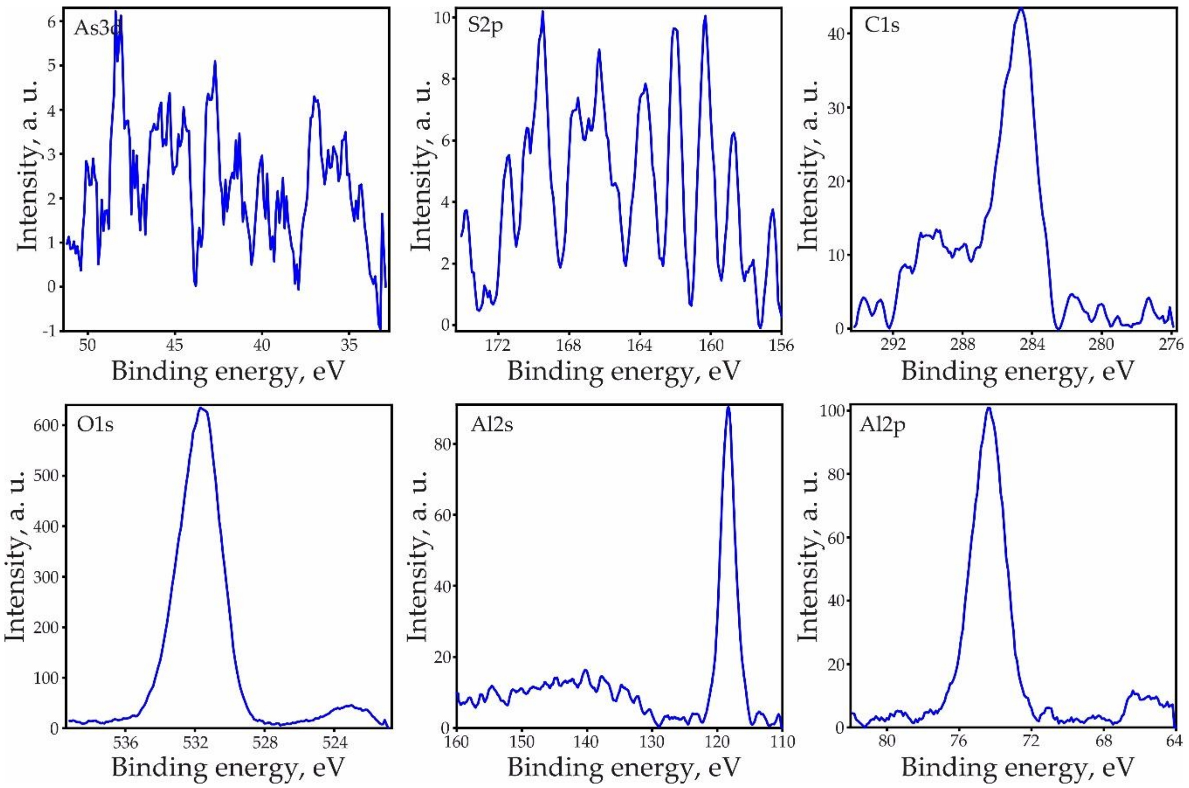

Figure S4. X-ray photoelectron spectra of porous aluminum oxide film formed in 0.6 M malonic acid solution at 100 mA·cm^{-2} current density with 2.79·10^{-1} g·L^{-1} arsenazo-I addition.

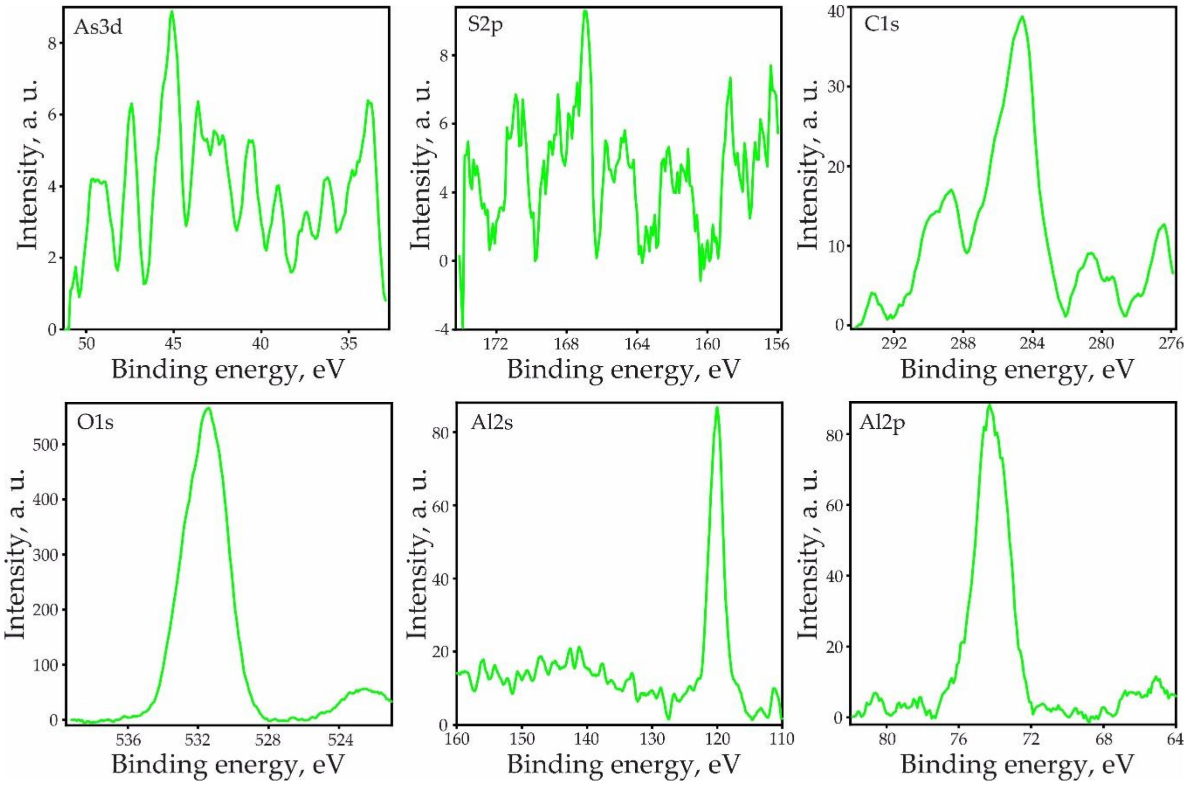

Figure S5. X-ray photoelectron spectra of porous aluminum oxide film formed in 0.6 M malonic acid solution at 100 mA·cm^{-2} current density with 2.0 g·L^{-1} arsenazo-I addition.

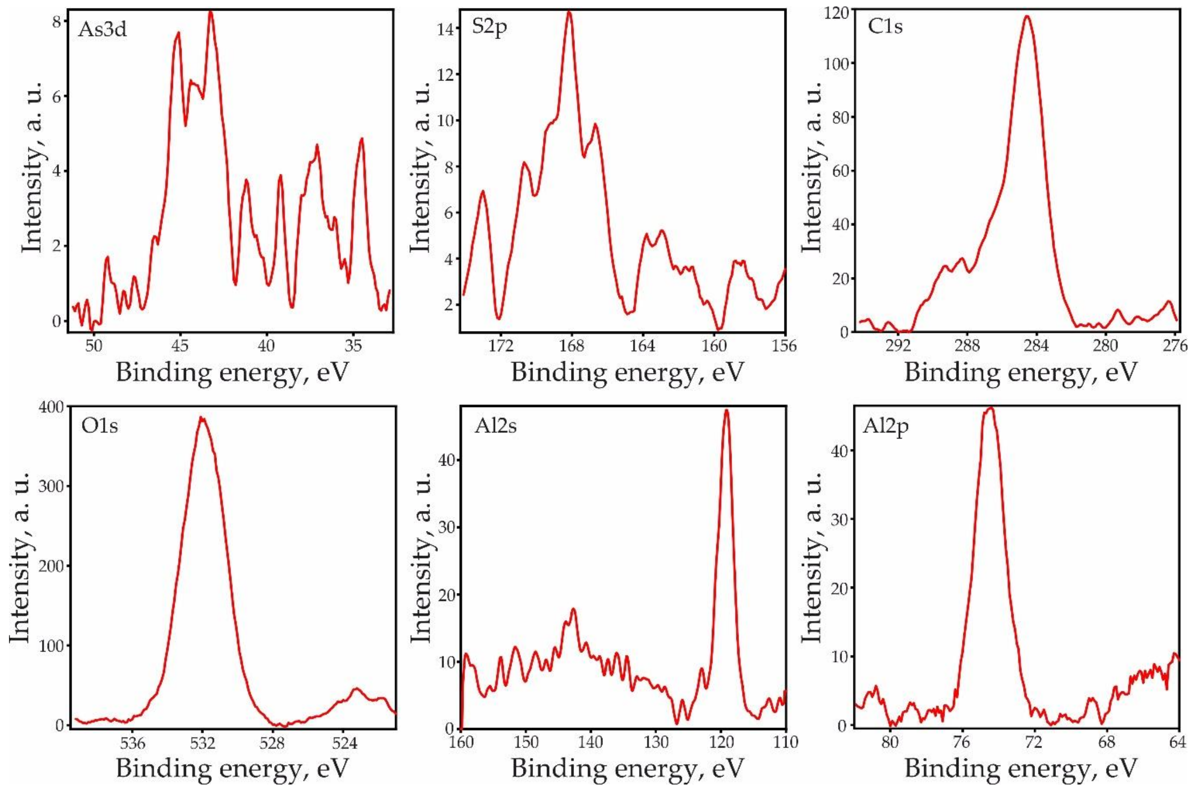

Figure S6. X-ray photoelectron spectra of porous aluminum oxide film formed in 0.6 M malonic acid solution at 100 mA·cm^{-2} current density with 3.72 g·L^{-1} arsenazo-I addition.

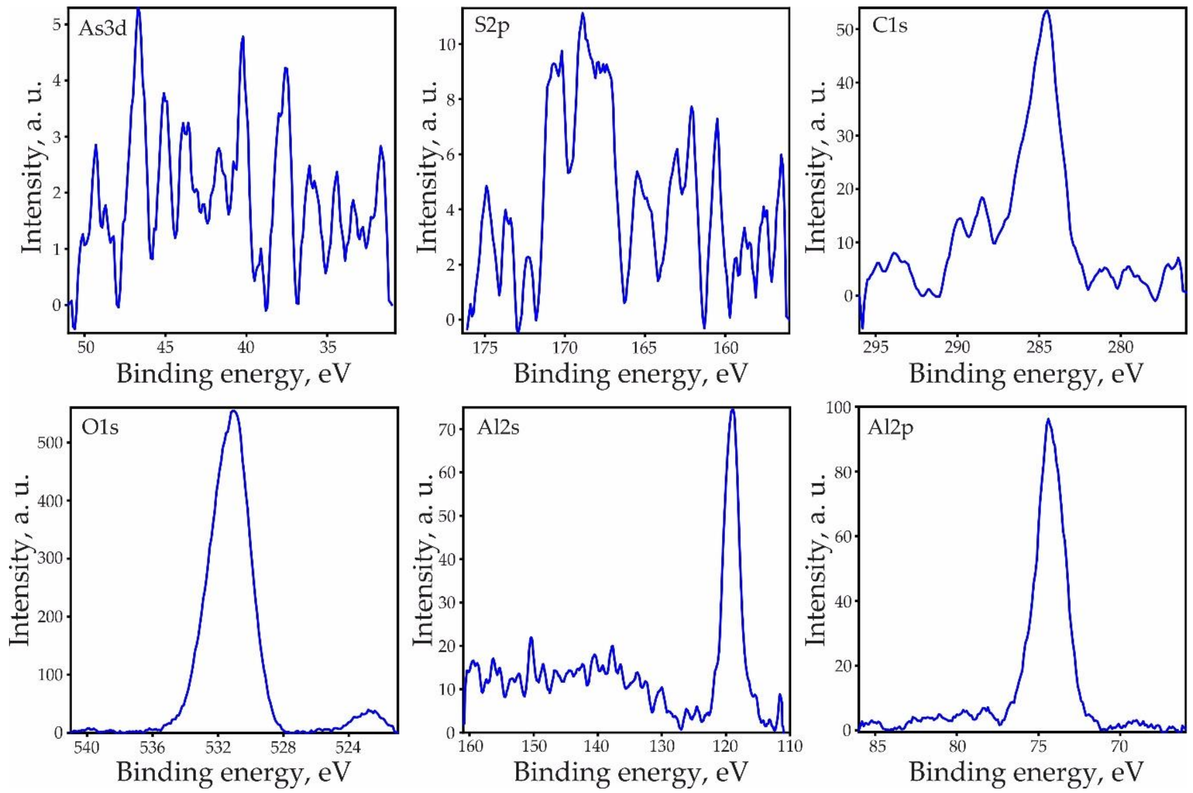

Figure S7. X-ray photoelectron spectra of porous aluminum oxide film formed in 0.6 M malonic acid solution at 200 mA·cm^{-2} current density with 1.90·10^{-1} g·L^{-1} arsenazo-I addition.

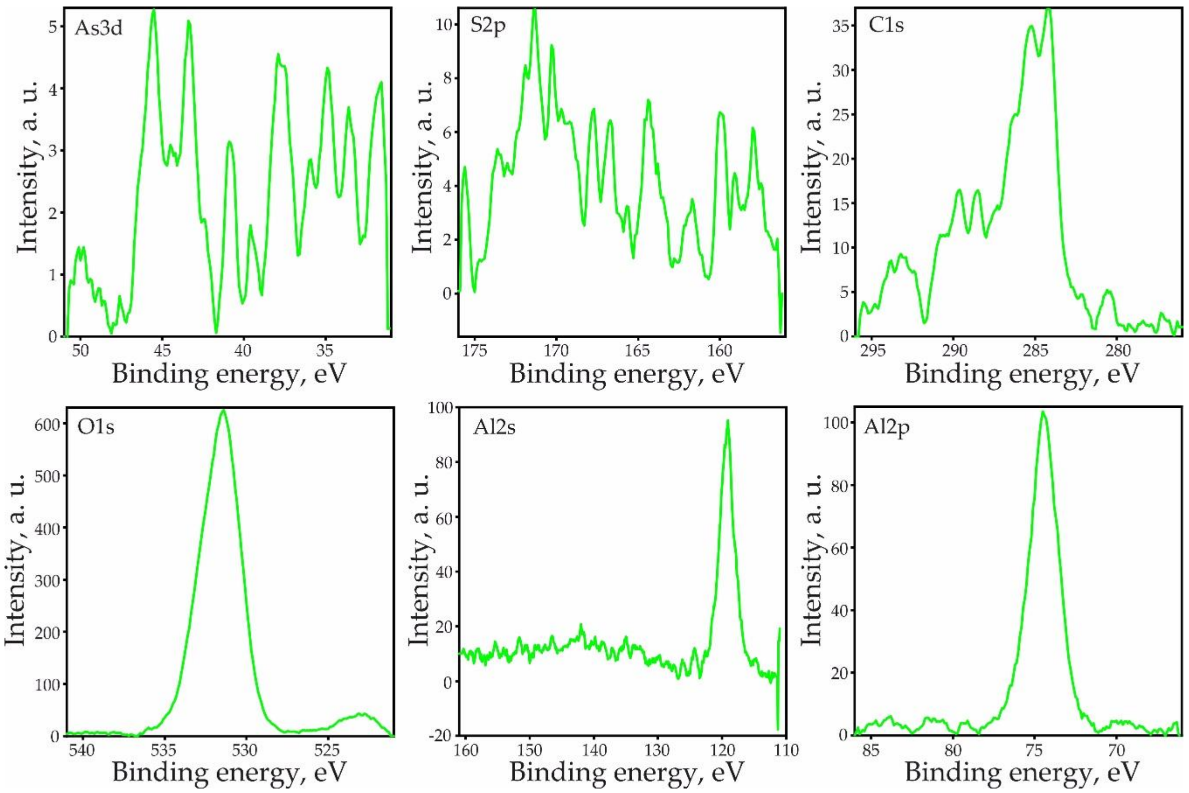

Figure S8. X-ray photoelectron spectra of porous aluminum oxide film formed in 0.6 M malonic acid solution at 200 mA·cm^{-2} current density with 2.0 g·L^{-1} arsenazo-I addition.

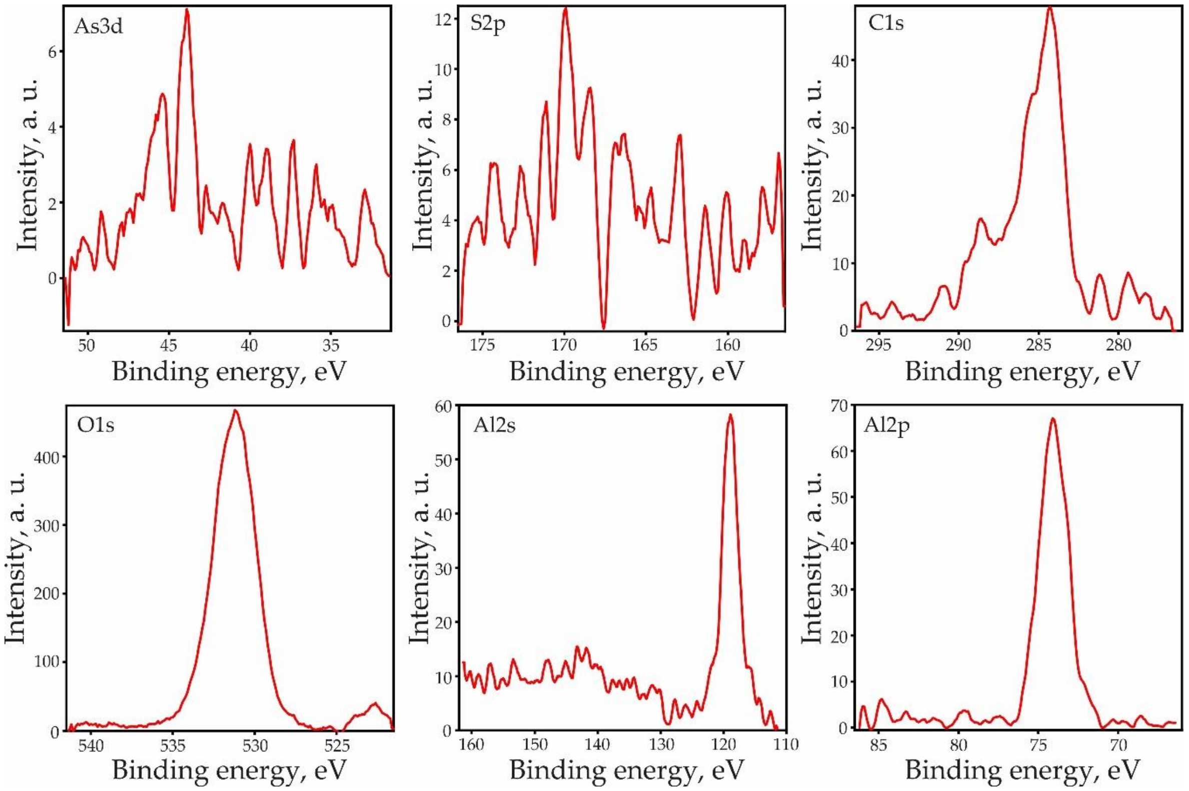

Figure S9. X-ray photoelectron spectra of porous aluminum oxide film formed in 0.6 M malonic acid solution at 200 mA·cm^{-2} current density with 4.0 g·L^{-1} arsenazo-I addition.

S2. Scanning Electron Microscopy of Porous Aluminum Oxide Films

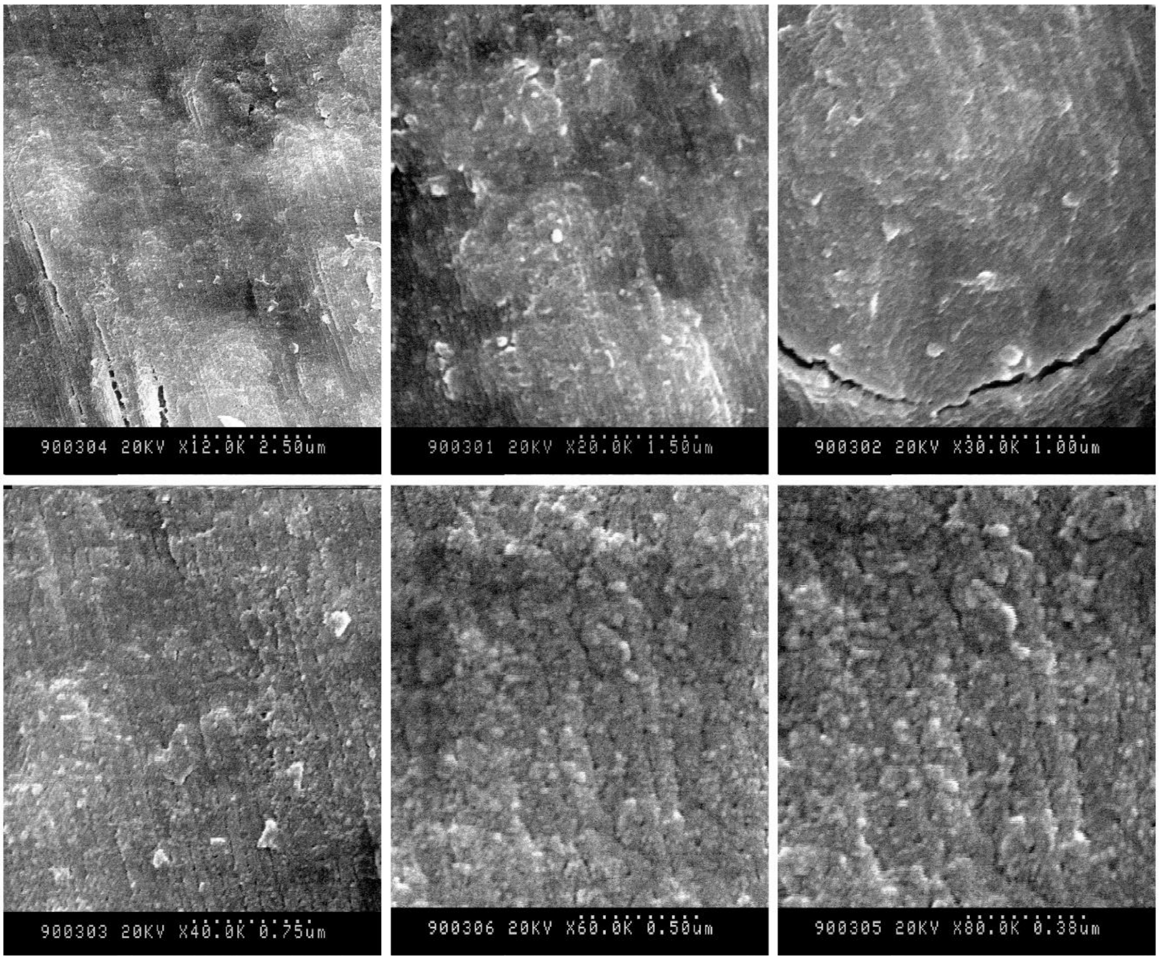

Figure S10. Surfaces of porous aluminum oxide film formed in 0.6 M malonic acid solution at 15.0 mA·cm^{-2} current density with 3.415 g·L^{-1} arsenazo-I addition.

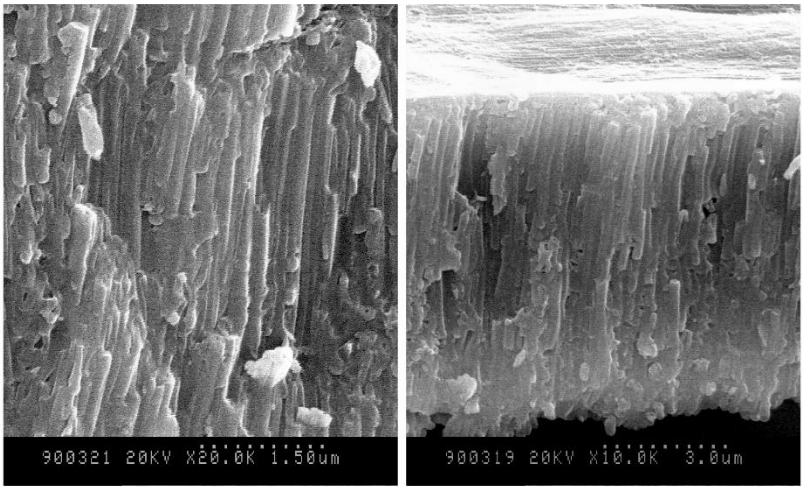

Figure S11. Cross-sections of porous aluminum oxide film formed in 0.6 M malonic acid solution at 15.0 mA·cm^{-2} current density with 3.415 g·L^{-1} arsenazo-I addition.

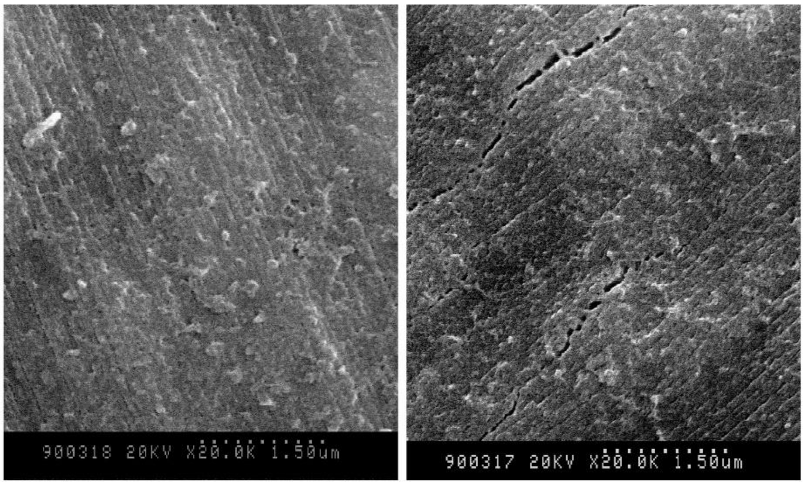

Figure S12. Surfaces of porous aluminum oxide film formed in 0.6 M malonic acid solution at 15.0 $mA \cdot cm^{-2}$ current density with 4.0 $g \cdot L^{-1}$ arsenazo-I addition.

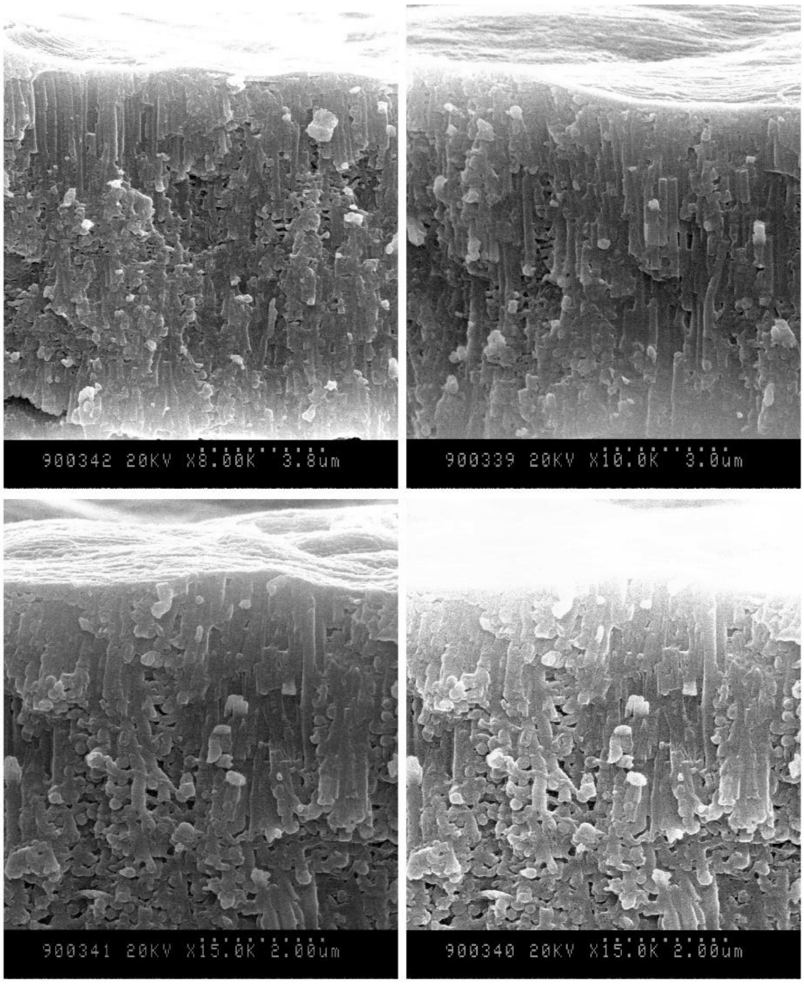

Figure S13. Cross-sections of porous aluminum oxide film formed in 0.6 M malonic acid solution at 15.0 $mA \cdot cm^{-2}$ current density with 4.0 $g \cdot L^{-1}$ arsenazo-I addition.

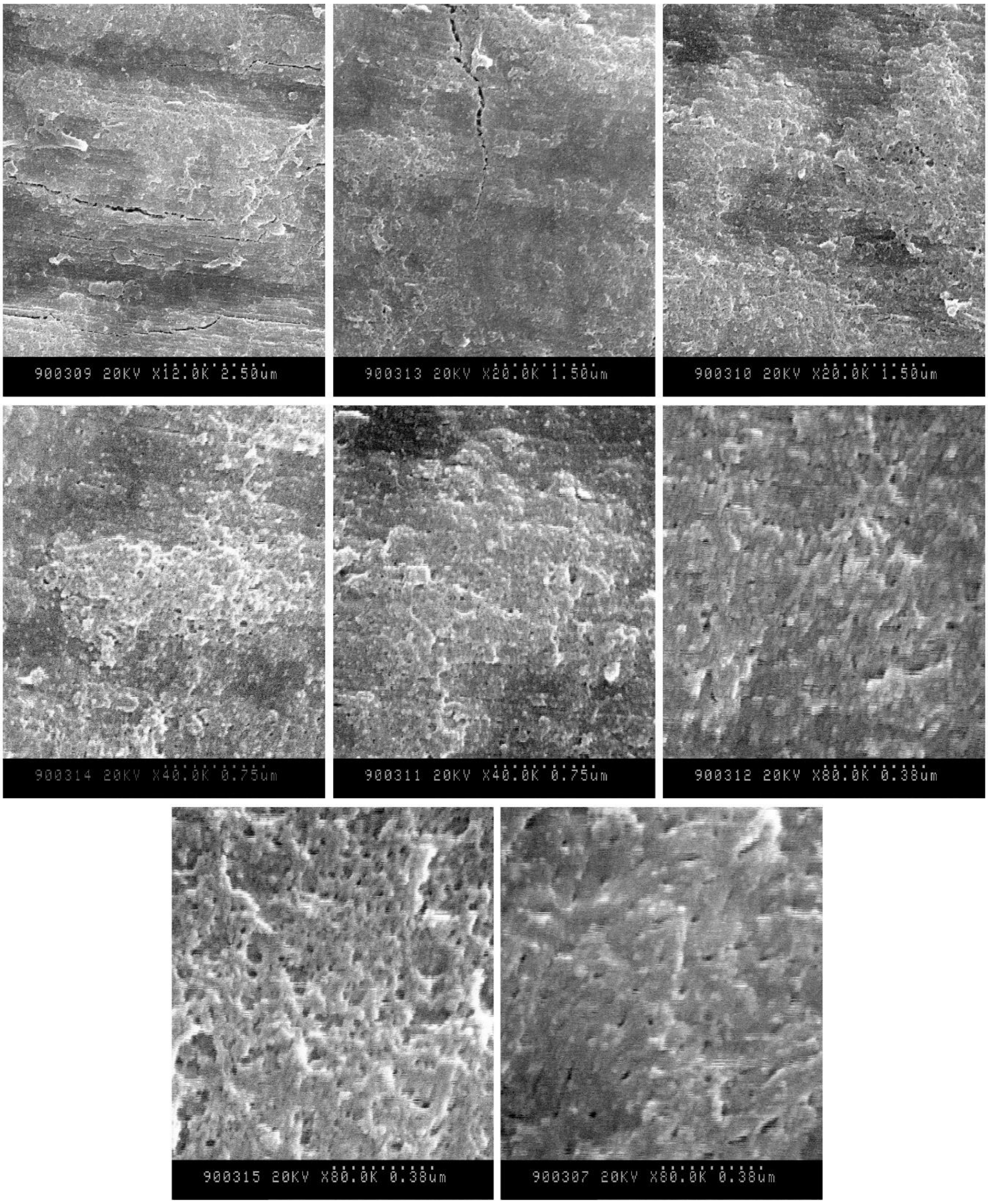

Figure S14. Surfaces of porous aluminum oxide film formed in 0.6 M malonic acid solution at 100 mA·cm^{-2} current density with 3.0 g·L^{-1} arsenazo-I addition.

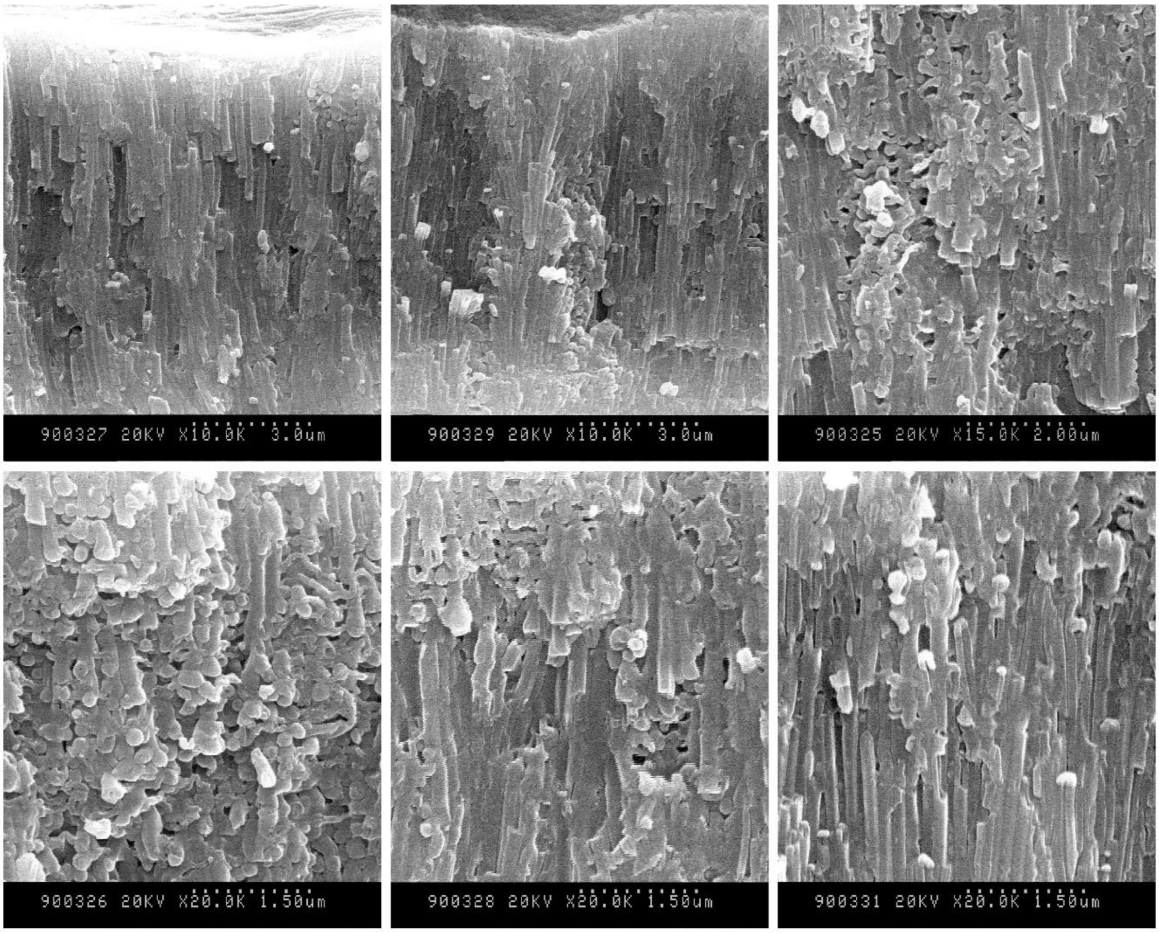

Figure S15. Cross-sections of porous aluminum oxide film formed in 0.6 M malonic acid solution at 100 mA·cm^{-2} current density with 3.0 g·L^{-1} arsenazo-I addition.

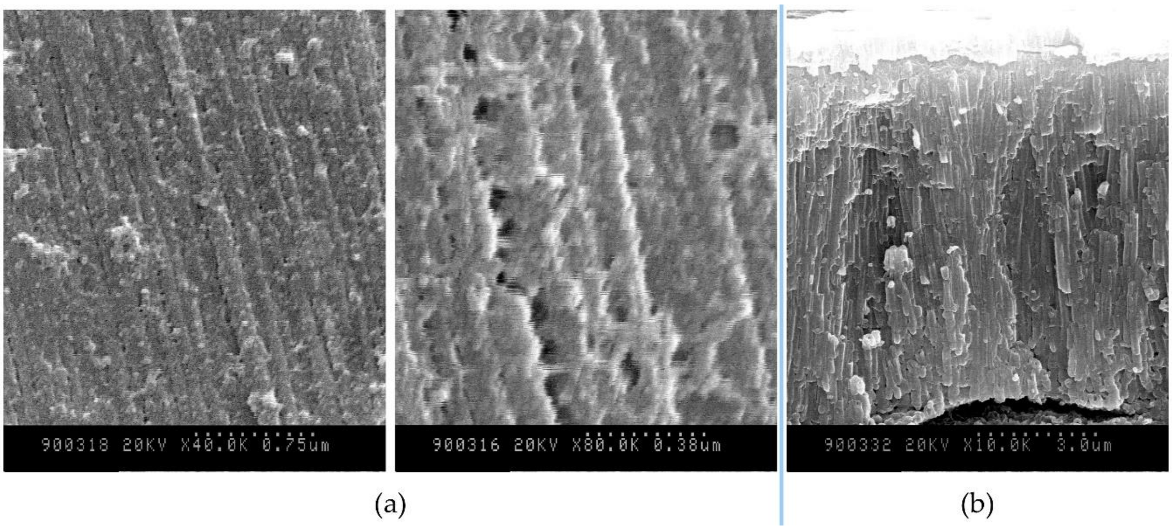

Figure S16. Surfaces (**a**) and cross-section (**b**) of porous aluminum oxide film formed in 0.6 M malonic acid solution at 100 mA·cm^{-2} current density with 3.721 g·L^{-1} arsenazo-I addition.

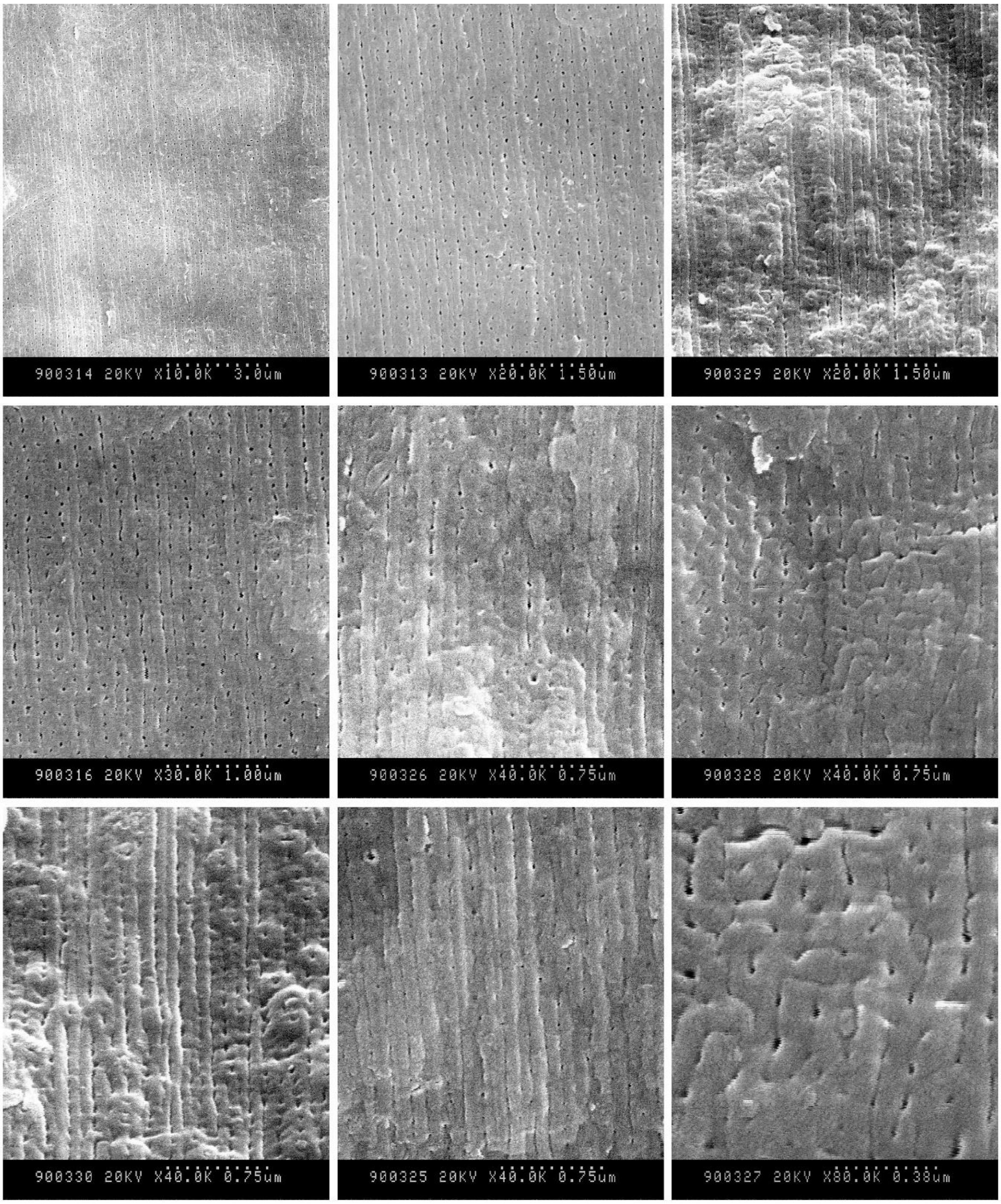

Figure S17. Surfaces of porous aluminum oxide film formed in 0.6 M malonic acid solution at 200 mA·cm^{-2} current density with 2.0 g·L^{-1} arsenazo-I addition.

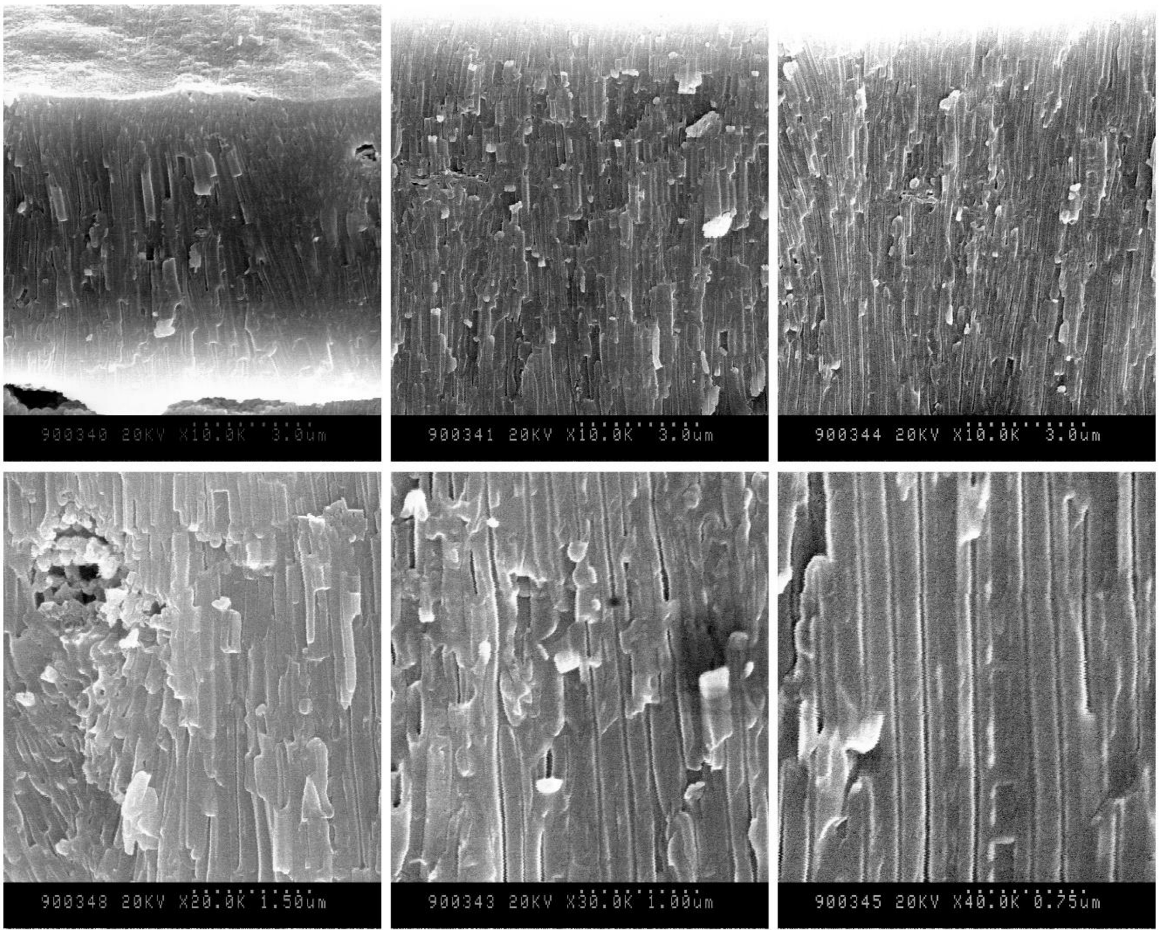

Figure S18. Cross-sections of porous aluminum oxide film formed in 0.6 M malonic acid solution at 200 mA·cm^{-2} current density with 2.0 g·L^{-1} arsenazo-I addition.

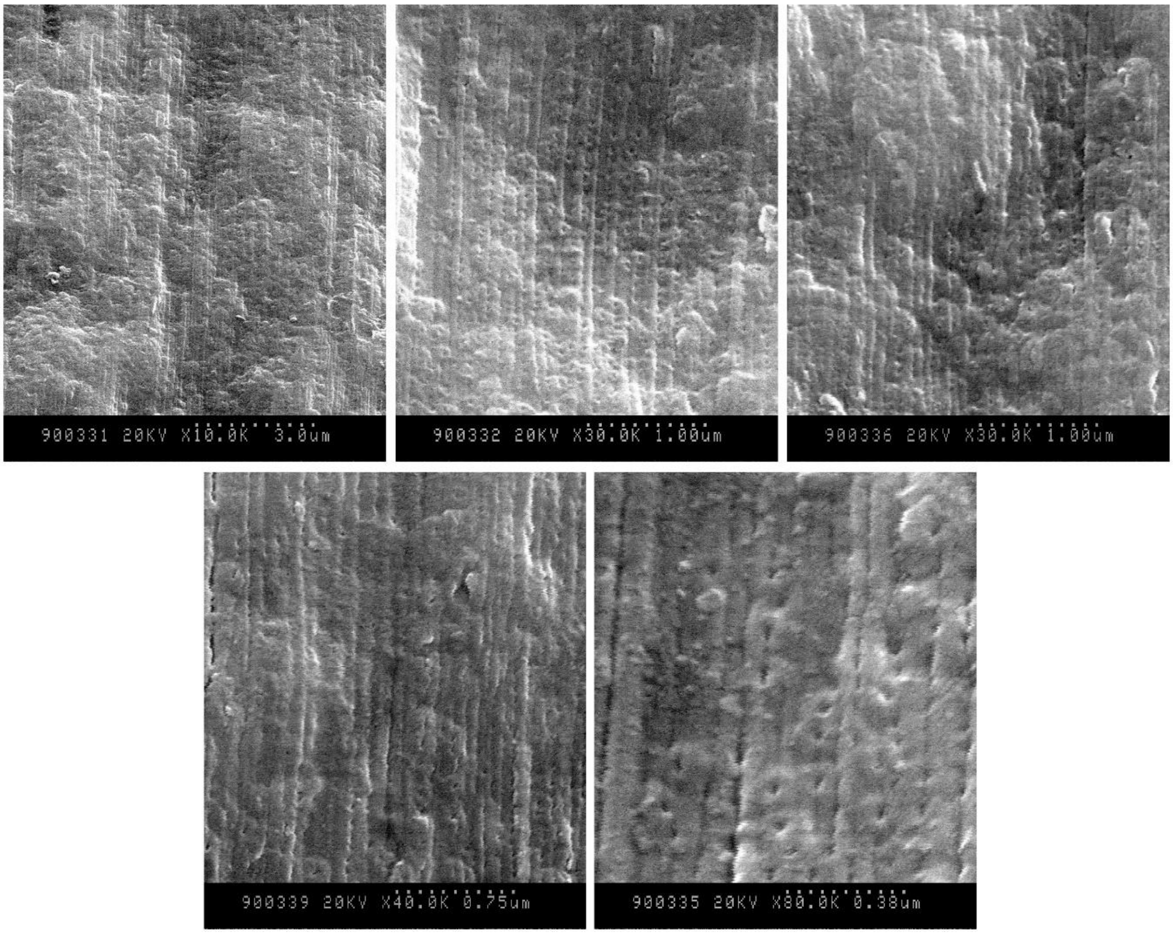

Figure S19. Surfaces of porous aluminum oxide film formed in 0.6 M malonic acid solution at 200 mA·cm^{-2} current density with 4.0 g·L^{-1} arsenazo-I addition.

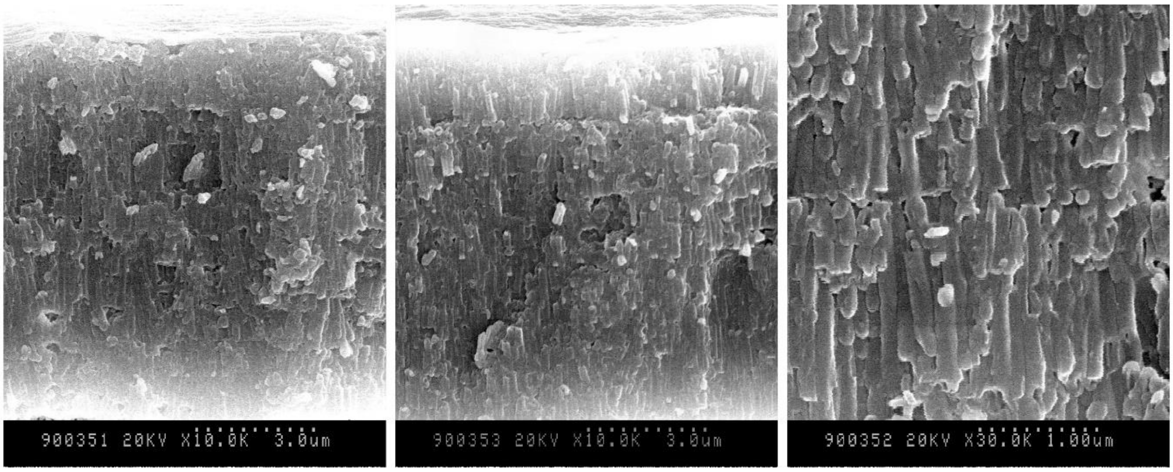

Figure S20. Cross-sections of porous aluminum oxide film formed in 0.6 M malonic acid solution at 200 mA·cm^{-2} current density with 4.0 g·L^{-1} arsenazo-I addition.
